# Role of Spectator Species for Amine-Surface Chemistry: Reactions of Amines and Alkenes on Pt(111)

**DOI:** 10.1021/jacs.5c00567

**Published:** 2025-05-12

**Authors:** Nils Brinkmann, Dave Austin, Bushra Ashraf, Duy Le, Talat S. Rahman, Katharina Al-Shamery

**Affiliations:** † Institute of Chemistry, 11233 Carl von Ossietzky University of Oldenburg, Carl-von-Ossietzky-Straße 9-11, 26129 Oldenburg, Germany; ‡ Department of Physics, 6243 University of Central Florida, Orlando, Florida 32816, United States

## Abstract

This study investigates the roles of ethylene and ethylidyne in the surface chemistry of *N*-methylaniline (NMA) on Pt(111). Using X-ray photoelectron spectroscopy, temperature-programmed desorption, and density functional theory calculations, we demonstrate that ethylidyne is not merely a passive spectator species but actively contributes to hydroamination. It facilitates C–N bond formation by transferring a methyl group to NMA, leading to the formation of *N*,*N*-dimethylaniline. Additionally, it stabilizes reaction intermediates and suppresses the decomposition of NMA. This works demonstrates, in contrast to the widely accepted notion, that ethylidyne is not just an inert spectator species; rather, it plays a dual role as both an active reaction partner and a stabilizer. In addition, the coadsorption of ethylene on an NMA-precovered surface shows a side reaction of ethylene with the decomposition products of NMA.

## Graphical abstract

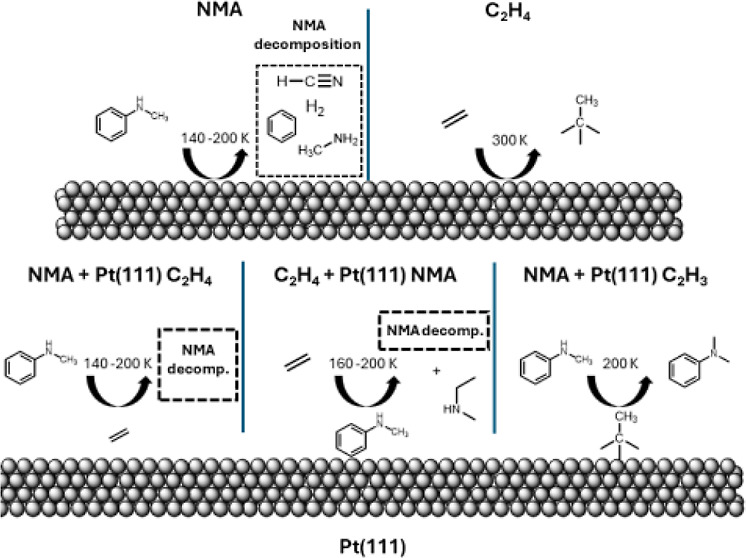

## Introduction

The industrial synthesis of amines is crucial for the production of pharmaceuticals, dyes, polymers, and various others chemicals, representing a multibillion-dollar market.^1,2^ The challenge lies in developing selective, efficient, and environmentally friendly synthesis routes. Amine synthesis is possible by different homogeneous catalysis methods, including nucleophilic substitution, reductive amination, hydroamination, cross-coupling, or hydroaminoalkylation, which are often based on metal complexes.^1,3–5^ However, the use of metal complexes needs high efforts for the separation from the product which is particularly important for all medical products needing a high-level of purity.^2,6–8^ The use of a heterogeneous catalyst in this context would be beneficial because it simplifies separation from the product and is more economic and robust.^6^

This study investigates the interaction between *N*-methylaniline (NMA) and ethylene on a platinum (Pt(111)) surface. The goal is to understand C–N bond formation for heterogeneous catalysts as an alternative to established homogeneous synthesis methods such as hydroamination or hydroaminoalkylation. Platinum is a promising candidate for catalyzing C–N bond formations due to its hydrogen affinity which facilitates N–H bond cleavage.^7^

However, the challenge for the development of heterogeneous catalysts is the high activation barrier because of electrostatic repulsion between the lone electron pair of the amine and the π-electron of the carbon–carbon double bond. That is why the development and examples are still rare, with few cases exhibiting the potential of supported metal nanoparticles (M = Pt, Pd, Au) and Pt surfaces for heterogeneously catalyzed hydroamination.^2,7–10^ Therefore, understanding the adsorption behavior of amines and alkenes on Pt surfaces is crucial.

The amine surface interaction is dependent on the chemical nature of the interface. Methylamines form aminocarbyne species by N–H and C–H bond activation, leading to decomposition products such as hydrogen cyanide. On the other hand, aromatic amines such as aniline tend to adsorb with the ring parallel to the surface facilitating C–N bond activation.^11–15^

Ethylene surface chemistry is well-known: ethylene adsorbs under ultrahigh-vacuum conditions at temperatures above *T* = 100 K via a di-σ-bounded state and forms a stable ethylidyne species when heated up to 240–320 K.^6,16–19^ With further heating, ethylidyne decomposes to several carbonaceous species via dehydrogenation, leading to coking of the surface above 400 K.^6,16^

The role of ethylidyne and its diverse carbonaceous species in the platinum-catalyzed hydrogenation of alkenes has been the subject of discussion.^15,17^ It is generally assumed that alkylidynes only act as spectator species but are not involved in the mechanism of alkene hydrogenation.^18–20^

The role of ethylidyne in other heterogeneously catalyzed reactions as for C–N bond formations has not been investigated so far and is the key topic of this study. The question is whether ethylidyne acts as a spectator or plays an active role in C–N bond formation. With the above background, the coadsorption of NMA and ethylene/ethylidyne on Pt(111) will be presented here in its entity by using surface-science techniques and density functional theory (DFT)-based calculations to get a detailed and fundamental understanding of the amine-surface chemistry.

## Results: *N*-Methylaniline and Ethylene on Pt(111)

The experiments were performed in a self-designed ultrahigh-vacuum (UHV) system with a base pressure below 1 × 10^–10^ mbar. The Pt(111) crystal was cleaned by argon sputtering and annealing. The cleanliness of the Pt(111) surface was checked by X-ray photoelectron spectroscopy (XPS) and temperature-programmed desorption (TPD). NMA and ethylene were dosed onto the platinum surface cooled by liquid nitrogen, using a pinhole doser. More details about the experimental setup and experimental details are given in the Supporting Information.

The adsorption behavior and surface chemistry of NMA and ethylene were investigated individually as reference for the coadsorption studies (for further details, see the Supporting Information sections 3 and 4). TPD spectra (see Figure S2) show that decomposition of NMA is predominant at low coverages starting with C–H bond activation at the methyl group and leads to hydrogen cyanide desorption. At higher coverages, NMA tends to desorb molecularly from the surface.^21^ DFT calculations revealed that this can be attributed to NMA lying flat on the surface at low coverages but with a tilting of the aromatic ring away from the surface at higher coverages. Thus, we used a multilayer coverage of NMA in experiments shown here. This multilayer coverage of NMA on Pt(111) and its thermal changes were investigated by XPS (see Figure S3), showing a coking of the surface above temperatures of 140 K.

Temperature-programmed XP spectra (see Figure 1) and detailed C 1s spectra at *T* = 108, 300, and 500 K (see Figure S4) were measured to follow thermal changes of ethylene adsorbed on Pt(111) between *T* = 120 K and *T* = 800 K.

**Figure 1:**
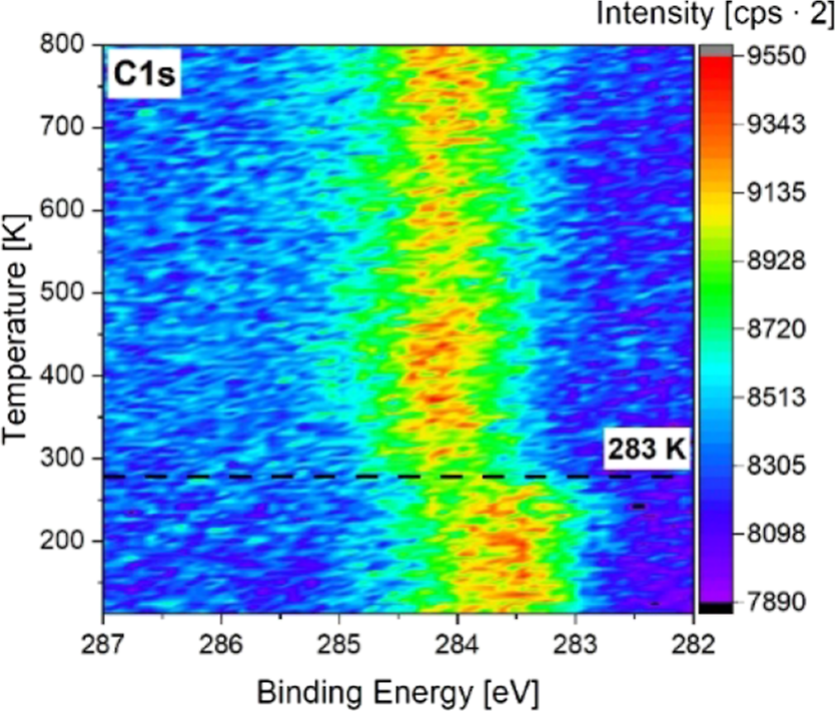
Temperature-programmed XP spectra of ethylene adsorbed at *T* = 110 K on Pt(111) in the temperature range from 120 K up to 800 K.

Ethylene exhibits one broad signal at 283.5 eV in the C 1s spectrum. The C1s signal shifts to 284.0 eV between *T* = 283 K and *T* = 300 K. This process begins at *T* = 283 K and finishes at 300 K. There is no decrease in the C 1s intensity or any further changes visible by heating up to 800 K. The results are in good accordance with the literature which reports ethylidyne formation at *T* = 290 K.^16^ The upshift of the binding energy at *T* = 283 K is due to the formation of ethylidyne on Pt(111) which is the surface species found above 300 K.^16,17,22^ The TP-XPS indicates a coking of the platinum surface by ethylidyne by further heating starting at 400 K up to 800 K. TPD spectra of ethylene are discussed in the Supporting Information (see Figure S5).

## Coadsorption Experiments: Ethylene/*N*-Methylaniline on Pt(111)

The coadsorption experiments were carried out in three ways. The first experiment started by dosing a monolayer ethylene first and subsequently performing the adsorption of NMA onto the ethylene-precovered surface. The coadsorption experiment was also performed in reverse order, so that ethylene was adsorbed on the NMA-precovered surface. The third coadsorption experiment includes the adsorption of NMA on an ethylidyne-precovered surface.

The results of the coadsorption experiments of ethylene and NMA in both orders (see Figures S6–S10) are only briefly summarized here and fully discussed in the Supporting Information (sections 5 and 6).

The coadsorption experiments of NMA and ethylene demonstrate that the ethylene-precovered surface behaves differently from the NMA-precovered surface. The ethylene-precovered surface shows similar results in TPD and XPS spectra as compared to those found for the adsorption of NMA and ethylene individually on Pt(111). The reverse order exhibited enhanced decomposition to HCN and methylamine on the NMA-precovered surface which desorb from the surface at *T* = 170 K. This low-temperature desorption feature in the TPD spectra was not present on the ethylene-precovered surface. Besides the formation of HCN and methylamine, the formation of ethyl methyl amine was indicated by the TPD spectra.

## Coadsorption Experiment: Ethylidyne/*N*-Methylaniline on Pt(111)

While the coadsorption of NMA and ethylene shows either no difference or a side reaction to ethyl methyl amine on the NMA-pre covered surface, the XP spectra in Figure 2 exhibit significant differences for the coadsorption of ethylidyne and NMA.

**Figure 2:**
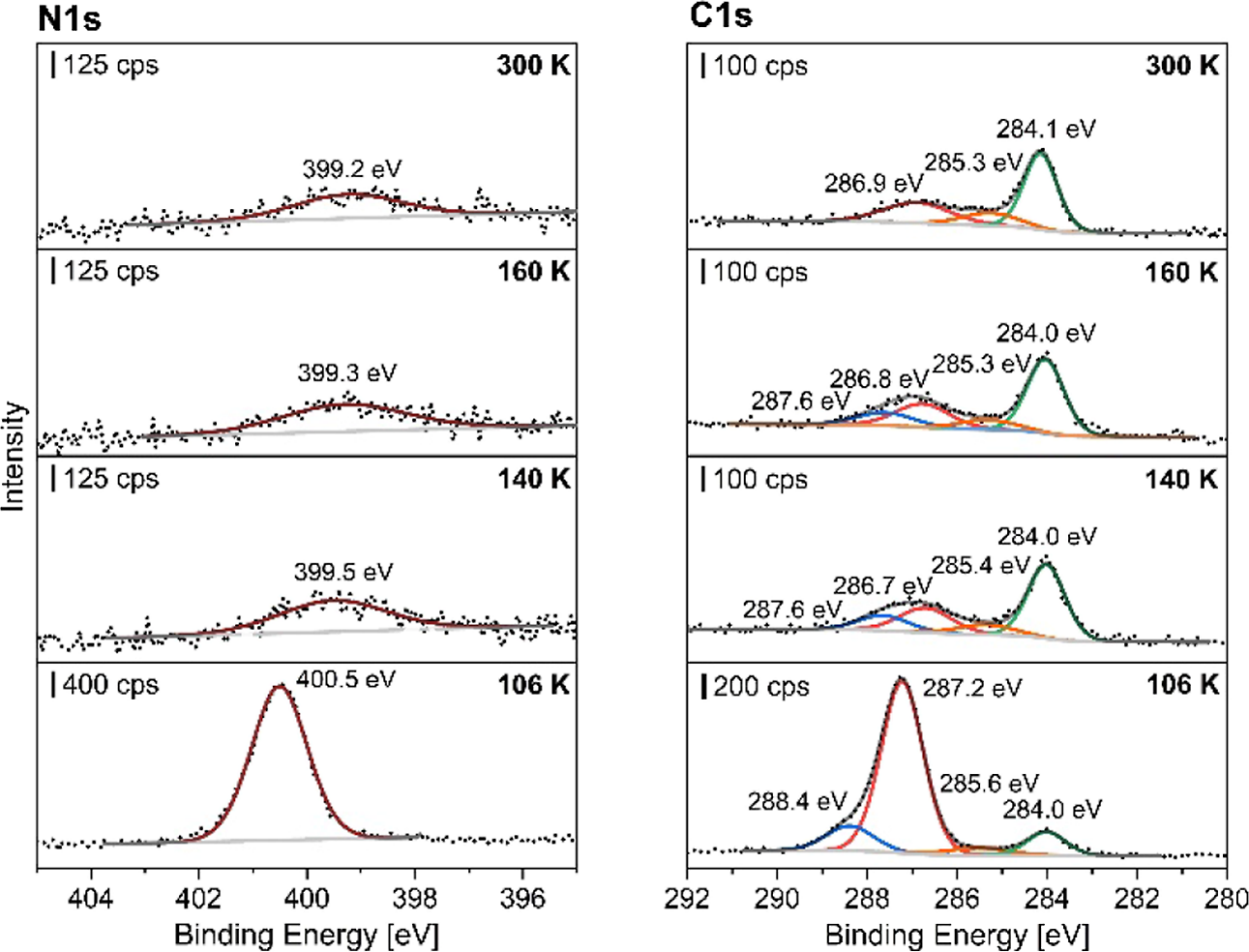
XP spectra of coadsorption of ethylidyne and *N*-methylaniline (NMA) at *T* = 106 K at Pt(111) followed by heating to the specified temperatures with the N 1s spectra (left) and the C 1s spectra (right). A monolayer ethylene was adsorbed first, and the ethylene-precovered surface was heated up to *T* = 298 K for 2 min to form ethylidyne. A multilayer NMA was then dosed on the ethylidyne-precovered surface at liquid nitrogen temperature. The Pt(111) single crystal was heated for 2 min to the elevated temperatures and cooled down before XP spectra were collected at *T* ≤ 110 K.

After exposure of the ethylidyne precovered platinum surface with NMA, one intense amine signal was observed at 400.5 eV in the N 1s spectrum, corresponding to the amine group of NMA.^21,23–25^ The phenyl ring and the methyl group of NMA lead to two signals at 287.2 and 288.4 eV with an appropriate intensity ratio of 6:1 in the C 1s spectrum. The signal at 284.0 eV corresponds to ethylidyne.^16,21^ An additional signal can be observed at 285.6 eV, indicating the formation of a new species that is not observed for NMA adsorption only.

By heating up to 300 K, the amine N 1s signal decreases significantly and downshifts by 1.3 eV. The amine signal remains present in contrast to the NMA/ethylene coadsorption experiments, indicating an increased stability of the amine. The downshift of the amine signal from 400.5 to 399.2 eV corresponds to a higher electron density of the amine, which can be due to a changed interaction with the platinum surface or chemical transformation of the amine by bond breaking and formation. The amine signal at 399.2 eV lies within the range of tertiary amines.^26,27^ The formation of a tertiary amine would also explain the formation of the additional species related to the peak at 285.6 eV which lies in the region for a possible C–N bond formation.^28,29^ Similar C 1s and N 1s values have been observed for 1,3,5- tris­(diphenylamino)­benzene.^27^

The C 1s signals also show a downshift by 0.5 and 0.8 eV for the NMA signals and by 0.2 to 285.4 eV for the additional peak when heating to *T* = 140 K. When heating up to *T* = 300 K, the high binding energy shoulder at 287.6 eV has vanished and a broader species at 286.9 eV is present in addition to the ethylidyne peak at 284.1 eV. The downshift is likely due to thermal changes by dehydrogenation and coking leading to C_*x*_H_*y*_ fragments of the remaining phenyl ring.^16,30–32^

TPD is used for the identification of the additional species and to gain further insights into reaction pathways and surface intermediates.

Table 1 shows the relevant mass fragments for the TPD coadsorption experiments.^33^ An overview of relevant mass fragments of NMA, ethylene, decomposition, and reaction products is given in Figure S8.

**Table 1:** List of Selected Mass Fragments (*m*/*z*) and Contributors for *N*-Methylaniline and Its Hydroamination, Hydroaminoalkylation, and Decomposition^33^ Products^a^

*m*/*z*	fragment	description
135	C_9_H_13_N^+^	*N*-ethyl-*N*-methylaniline
121	C_8_H_11_N^+^	*N*,*N*-dimethylaniline
120	C_8_H_10_N^+^	*N*-ethyl-*N*-methylaniline/*N*,*N*-dimethylaniline
106	C_7_H_8_N^+^	*N*-methylaniline
92	C_6_H_5_NH^+^	aniline
77	C_6_H_5_^+^	benzene
28	N_2_^+^/CO^+^/C_2_H_4_^+^	nitrogen, carbon monoxide, ethylene
27	HCN^+^/C_2_H_3_^+^	hydrocyanic acid, ethylene
26	CN^+^/C_2_H_2_^+^	cyanide, ethylene

a The fragmentation pattern was obtained from NIST database.

The coadsorption TPD spectrum for the ethylidyne-precovered surface in Figure 3 exhibits three desorption features at *T* = 200 and *T* = 210 K and a shoulder at *T* = 229 K for the mass fragment *m*/*z* = 106 [C_7_H_8_N^+^]. The desorption features at *T* = 210 and 229 K were associated with the NMA multilayer and monolayer desorption, respectively, in a previous study and are in good accordance with aniline on Pt(111).^11,21^

**Figure 3:**
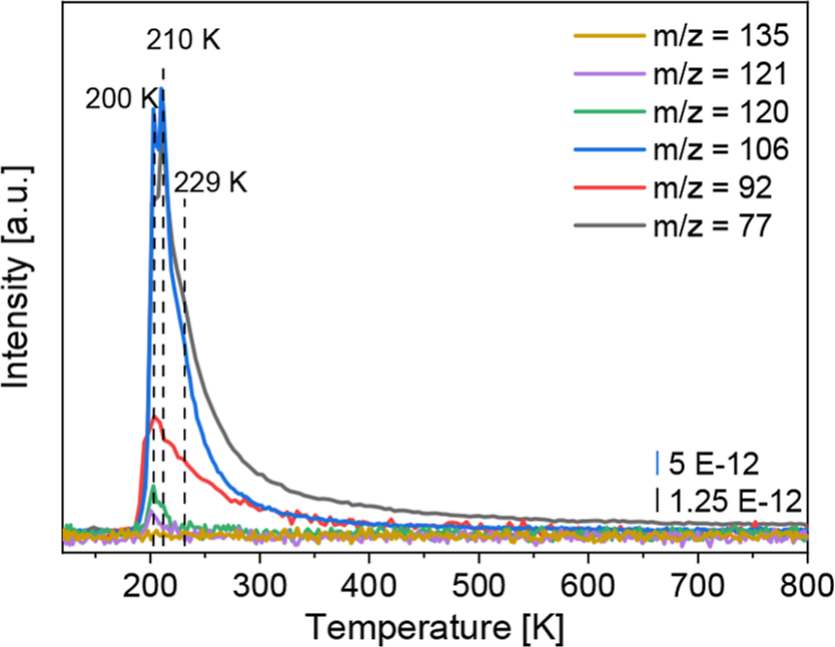
TPD spectrum of ethylidyne/NMA on Pt(111). A monolayer of ethylene was dosed first onto the surface. Ethylidyne was formed by heating the crystal to *T* = 298 K for 2 min. A multilayer of NMA was then dosed onto the ethylidyne-precovered surface at liquid nitrogen temperature. The blue curve has a four times higher intensity and was scaled down for better visibility.

In addition to the NMA desorption signals, the desorption feature at *T* = 200 K is due to product formation involving C–N bond formation as further higher mass fragments were detected. Possible hydroamination products would be *N*-ethyl-*N*-methylaniline (NEMA) and the possible hydroaminoalkylation product *N*-propylaniline (NPA). They can be identified from recording the mass fragments *m*/*z* = 135 [C_9_H_13_N^+^], 121 [C_8_H_11_N^+^], and 120 [C_8_H_10_N^+^]. While both products would exhibit the mass fragment *m*/*z* = 135, only NEMA would have mass fragments *m*/*z* = 121 and 120 in contrast to NPA. However, no mass fragment *m*/*z* = 135 was observed so that both stated products can be ruled out.

Another possibility neglected so far is the addition of the methyl group of the ethylidyne via hydroamination to NMA, leading to the formation of *N*,*N*-dimethylaniline (NDMA). The fragmentation pattern of NDMA exhibits the mass fragments *m*/*z* = 121 [C_8_H_11_N^+^], 120 [C_8_H_10_N^+^], and 92 [C_6_H_5_NH^+^],^33^ which fits to the measured TPD spectra in this work. However, the question arises whether the mass fragment *m*/*z* = 92 is only due to the formation of *N*,*N*-dimethylaniline or also from the formation of aniline. However, aniline formation can be excluded here since aniline formation would not lead to a detection of the benzene mass fragment *m*/*z* = 77 [C_6_H_5_^+^]. Furthermore, the mass fragment *m*/*z* = 66 (not shown), typical for aniline, is missing.^34^ Therefore, the desorption peak at *T* = 200 K is solely attributed to the formation of *N*,*N*-dimethylaniline (NDMA). The TPD spectra of the mass fragments for ethylene and decomposition products *m*/*z* = 28 [N_2_^+^/CO^+^/C_2_H_4_^+^], 27 [HCN^+^/C_2_H_3_^+^], and 26 [CN^+^/C_2_H_2_^+^] are shown in the Supporting Information (Figure S9) and discussed there in the context of the coadsorption experiments with NMA and ethylene. The TPD spectra confirm the trend seen in XPS of a stabilized amine, as less decomposition to HCN is apparent in comparison to ethylene/NMA coadsorption.

## Results of DFT Calculations

Here, DFT calculations study the process and energy dynamics of forming *N*,*N*-dimethylaniline from NMA molecules that are densely packed on the Pt(111) surface. It delves into the stability of NMA at high coverage, emphasizing the role of dehydrogenation in heightening the reactivity of the nitrogen atom, and influences the density of states (DOS) for the nitrogen and phenyl ring, resulting in changes in electronic structure and interaction with the platinum surface. This heightened reactivity is pivotal for creating *N*,*N*-dimethylaniline, facilitating interactions between NMA and ethylidyne.

The DFT calculations were carried out using the Quantum ESPRESSO package^35^ with the GGA-PBE^36^ exchange–correlation functional and DFT-D3 ^37^ van der Waals correction. A plane-wave cutoff of 50 Ry and a charge density cutoff of 500 Ry were used. A 5 × 5 × 1 Monkhorst–Pack^38^ *k*-point mesh was employed for both relaxation and DOS calculations, with a Gaussian smearing of 0.05 eV. Additional computational parameters and convergence details are provided in the Supporting Information (section 2).

The investigation of *N*,*N*-dimethylaniline formation was conducted at high coverage because of the findings on the coverage-dependent interaction of NMA in a previous study,^21^ indicating NMA decomposition at low coverages. The calculations here demonstrate that at high levels of coverage, the NMA molecule has the potential for dehydrogenation as the dehydrogenation of the methyl and amine groups results in an energy reduction of −280 and −160 meV, respectively. Our emphasis is on the dehydrogenation of the amine group as it triggers an increase in the reactivity of the nitrogen atom, which is crucial for the formation of *N*,*N*-dimethylaniline. This heightened activity in the nitrogen atom is evident in Figure 4a, where an increase in the DOS is observed both below the Fermi level for the nitrogen’s p_*z*_ and p_*x*_ orbitals and above the Fermi level with a peak in the p_*z*_ DOS at approximately 800 meV.

**Figure 4:**
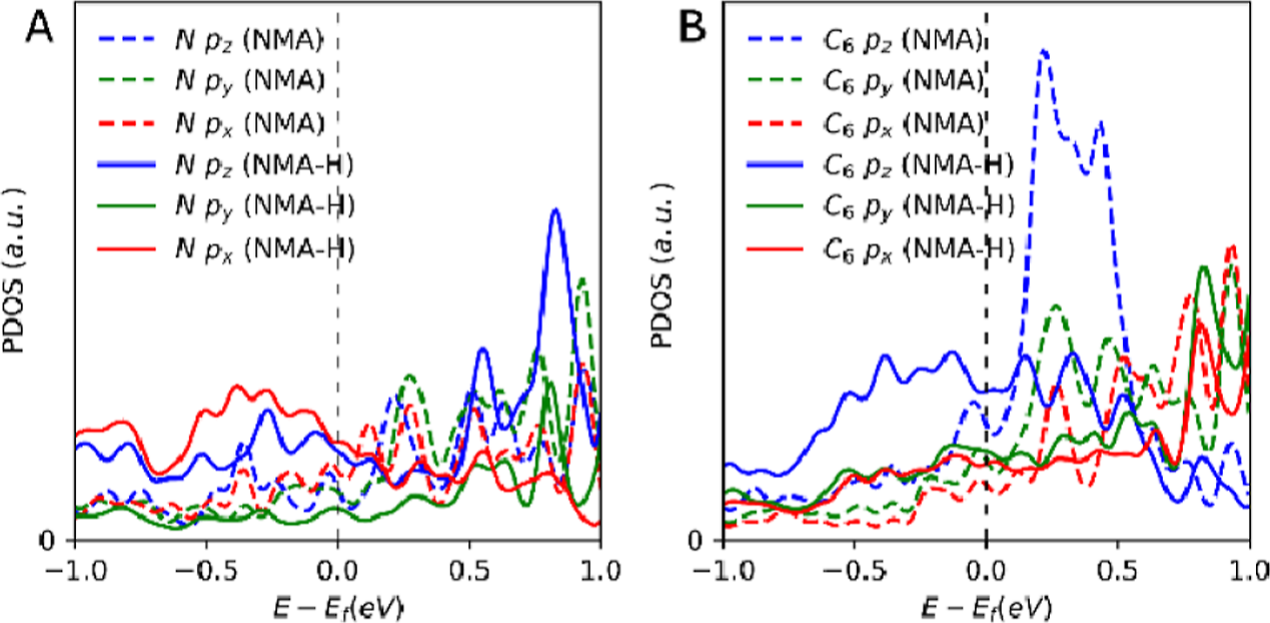
Orbital projected DOS for the NMA (dashed lines) and the dehydrogenated NMA (solid line) molecule. The p_*z*_, p_*y*_, and p_*x*_ are blue, green, and red, respectively. (A) Projection for the nitrogen atom and the (B) phenyl ring.

The strong hybridization with the surface causes delocalization of the p orbitals of the nitrogen. An interesting reconfiguration of the NMA molecule can be seen after this dehydrogenation. Figure 4b shows the projected DOS of the phenyl ring in both the dehydrogenated and hydrogenated form. Before the removal of the hydrogen from the amine group, there is a more localized p_*z*_ in the conduction region. After the hydrogen is removed, in this 2 eV range centering around the Fermi edge, a broad peak can be seen coming from the p_*z*_ of the phenyl ring. This delocalization points to the phenyl ring interacting with the platinum surface, which can be confirmed by the charge difference plots in Figure 5. The green indicates charge depletion, and the brown indicates charge accumulation. In Figure 5a, there is much more charge redistribution between the phenyl ring and the surface for the dehydrogenated NMA. This increase in the interaction between the phenyl ring and the surface also explains why breaking the N–H bond is more difficult in the high coverage than that in the low coverage regime. In Figure 5b, we see that when the NMA is adsorbed on the platinum surface, most of the charge moves away from the nitrogen hydrogen bond. The weakening of this bond explains why activation of the N–H bond is more accessible in this high coverage vs the low coverage regime and why the focus here is on this bond.

**Figure 5:**
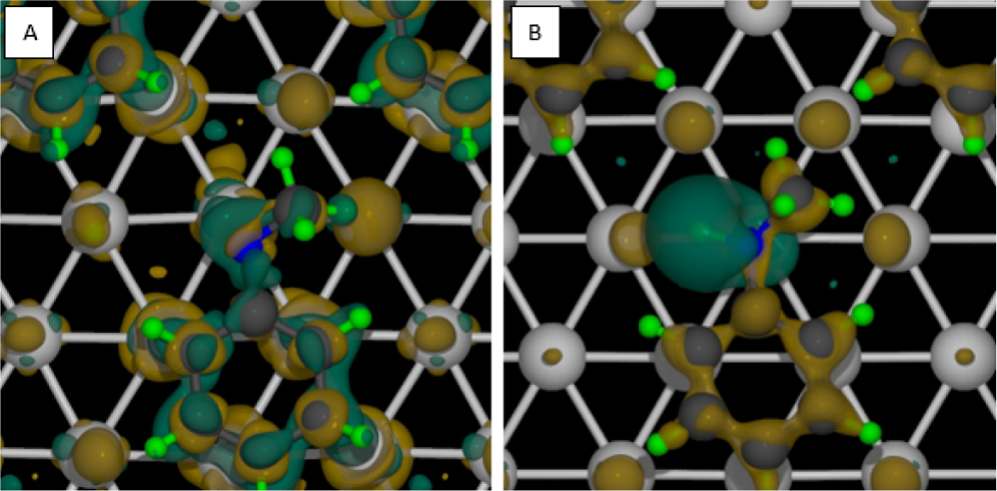
Charge difference for the (a) dehydrogenated NMA and the (b) NMA molecule. Green shows where the charge is moving from, and brown shows where the charge is moving to.

This redistribution of charge not only influences the strength of the N–H bond but also plays a crucial role in facilitating subsequent reactions. In particular, activation of the N–H bond is a key step in the formation of *N*,*N*-dimethylaniline, as illustrated in Figure 6. Two potential pathways for this transformation exist. In the first pathway, ethylidyne interacts with the NMA amine hydrogen, requiring a high energy input of 2.55 eV. In the second pathway, dehydrogenation of the amine group occurs first, increasing energy by only 0.32 eV and significantly lowering the energy barrier for ethylidyne binding to nitrogen (0.72 eV). The final step—ethylidyne breaking and forming a CH species along with *N*,*N*-dimethylaniline—results in an energy decrease of 0.25 eV.

**Figure 6:**
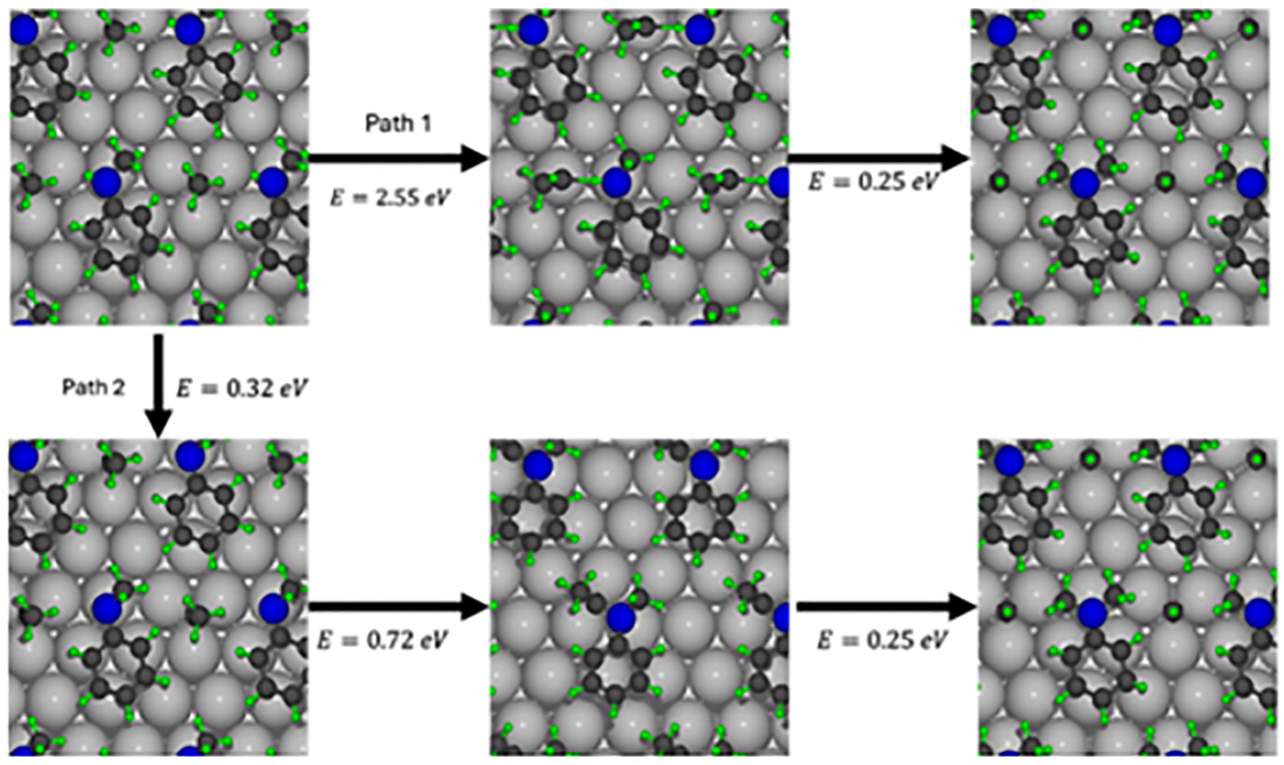
Pathways for the formation of dimethylaniline in two different paths. The first path, starting in the upper left corner and going to the right, shows the path from the NMA molecule. The second path, starting in the same place but going downward, shows the path with the dehydrogenated NMA molecule.

## Discussion

This study aimed to perform a UHV model study on the coadsorption of an aromatic amine with ethylene and ethylidyne at a Pt(111) surface to gain knowledge about the potential of the platinum surface for heterogeneously catalyzed C–N bond formation reactions and to elucidate the role of carbonaceous deposits on the surface chemistry of NMA.

The coadsorption experiments of NMA and ethylene demonstrate that the ethylene-precovered surface behaves differently from the NMA-precovered surface. While the ethylene-precovered surface showed no difference to the separate adsorption of NMA and ethylene, the NMA-precovered surface exhibited the formation of ethyl methyl amine which results from the reaction of ethylene with methylamines, a follow-up reaction from the partial decomposition of the NMA on the platinum surface at elevated temperatures.

A circumstance that can hinder a reaction of NMA at the ethylene-precovered surface is the formation of a strong interacting di-σ-bounded ethylene species.^12,13^ However, high adsorbate mobility is a key factor for heterogeneously catalyzed reactions for reaching the favorable adsorption sites as shown for the ethylene hydrogenation.^37^ Results from the literature demonstrate that the surface mobility and thus also the catalytic reaction can come to a standstill by coadsorption with a molecule that interacts strongly with the surface.^37^

The strongly interacting di-σ bonding complex is activated to ethylidyne above 240 K,^17–19^ which offers a high surface mobility because of a low energy barrier between the 3-fold fcc and hcp sites.^39^ However, the NMA decomposition starts at even lower temperatures at *T* = 140 K,^21^ leading to carbon residues remaining at the surface making a C–N forming reaction unlikely under these conditions, but when starting on an ethylidyne-precovered surface, the amine surface chemistry changes.

### Role of Ethylidyne on NMA Surface Chemistry

The role of ethylidyne in the hydrogenation of ethylene has been widely discussed in the literature. While there has been some considerations of ethylidyne as a participant in the hydrogenation of ethylene, nowadays the role of ethylidyne as a nonreactive spectator species is widely accepted.^17,20,40^ Therefore, one might expect that ethylidyne acts as a spectator molecule, blocking the fcc 3-fold hollow sites at the platinum surface which can hinder molecules from reaching the metal surface at high coverages.^39^ However, from the results of the coadsorption study presented here, a general interpretation of ethylidyne only as a nonreactive spectator species must be questioned since the coadsorption experiments show the formation of *N*,*N*-dimethylaniline by methyl group transfer on the ethylidyne-covered surface. Besides the hydroamination product, ethylidyne inhibits the decomposition of NMA, as apparent from TPD and XP spectra.

DFT calculations suggest that a potential reaction pathway for the formation of *N*,*N*-dimethylaniline could involve the abstraction of hydrogen from the nitrogen atom of NMA followed by the transfer of a methyl group of ethylidyne to NMA. The activation of the N–H bond and hydrogen abstraction by the platinum surface can occur preferentially at high coverages of NMA as DFT calculations have shown. Possible hydrogen abstraction by ethylidyne and formation of ethylidene can be ruled out as the comparison of the total energy of the formed intermediate state with the initial state of NMA and ethylene coadsorbed on the platinum surface shows an increase of the energy by 2.55 eV. The above indicates that this pathway is not thermodynamically favored. After hydrogen abstraction by the platinum surface, DFT calculations suggest that ethylidyne could interact with the nitrogen atom of NMA followed by transfer of the methyl group, formation of *N*,*N*-dimethylaniline, and coking of the surface. Thus, theory and experiment show that contrary to the interpretation in the literature, ethylidyne actively participates in the reaction and is therefore not just an uninvolved spectator but also has a stabilizing effect. By reducing the free-surface area, the aromatic ring with a larger space requirement cannot be adsorbed in parallel to the surface at high coverages, which decreases the interactions between ring and surface. This is in accordance with the literature exhibiting that alkylidyne layers weaken the adsorption of alkenes, which facilitates the hydrogenation of alkenes.^20,40,41^

### Heterogeneous Catalyst as an Alternative to Homogeneous Catalysts for C–N Bond Formation Reactions

The Pt(111) surface is used as a model catalytic surface for a widely used platinum catalyst. The question arises to what extent a heterogeneous Pt catalyst can be an alternative to homogeneous catalysts for different ways of C–N bond formation types such as hydroamination, hydroaminoalkylation, or cross-coupling.

The metal surfaces of heterogeneous catalysts do not seem to be suitable for hydroaminoalkylation reactions, in general. The main challenge is that α-H and β-H eliminations compete on metal surfaces, while the β-H elimination is favored especially at low temperatures.^18,19^ However, the α-H elimination is a crucial step in the homogeneously catalyzed hydroaminoalkylation at titanium complexes.^42,43^

The cross-coupling of different amines can be heterogeneously catalyzed by platinum nanoparticles supported by γ-Al_2_O_3_.^44^ The cross-coupling requires the formation of an imine by hydrogen abstraction, which then is converted with an amine in a second step and rehydrogenated to form the desired amine.^44^ In principle, platinum seems to be a promising candidate because of its hydrogen affinity, but the cross-coupling of amines is a structure-sensitive reaction, which requires coordinatively unsaturated Pt atoms with a moderate electron density. XPS spectra did not show any formation of an imine-like species, which is crucial for amine coupling. Previous studies have shown that coadsorbed oxygen can promote the formation of an imine species on Pt(111) so that amine cross-coupling could be feasible in the presence of oxygen under UHV conditions.^21^

Hydroamination encounters challenges due to repulsive forces between the alkene and the lone pair of the nitrogen atom. Theoretical calculations indicate that the platinum surface might mitigate this issue by reducing the electron density from the nitrogen atom at high coverages. However, this approach hinges on the amine remaining intact and ethylene not to strongly interacting with the platinum surface.

## Conclusions

To conclude, we have highlighted the role of ethylidyne on the amine-surface chemistry, showing that ethylidyne cannot be considered a nonparticipating spectator species; rather, it actively participates in the amine-surface chemistry by the formation of *N*,*N*-dimethylaniline. This is in contrast to the general assumption that alkylidynes act only as spectator species on metal surfaces. Furthermore, by combining theory and experiment, we demonstrated that N–H bond activation is the key to forming hydroamination products. The major challenge in hydroamination is the repulsive interaction between the electron-rich alkene and the lone pair of the nitrogen atom, leading to high activation barriers. The platinum surface reduces the charge at the nitrogen atom at high coverages, which is a key for hydroamination reactions, but the premise for a C–N bond formation is that the amine does not decompose and ethylene does not strongly interact with the platinum surface. Our findings highlight the importance of carbonaceous species on catalytic reactions, and future studies can explore the possibility of using longer alkylidynes for C–N bond formation reactions.

## Supplementary Material



## Notes

Experimental details, computational details, *N*-methylaniline on Pt(111): fragmentation pattern *N*-methylaniline, TPD spectra of an adsorbed submonolayer (150 s) and a multilayer (450 s) of *N*-methylaniline at Pt(111), XP spectra of an adsorbed multilayer of *N*-methylaniline (NMA) at *T* = 103 K at Pt(111), ethylene on Pt(111): XP spectra of C 1s region for an adsorbed monolayer of ethylene at *T* = 108 K at Pt(111), TPD spectra of ethylene adsorbed at Pt(111), XPS analysis of coadsorption experiments with ethylene/NMA on Pt(111): XP spectra of coadsorption of a monolayer ethylene and a multilayer *N*-methylaniline (NMA) at *T* = 103 K on Pt(111), TPD spectra of NMA and ethylene at Pt(111), TPD analysis of coadsorption experiments: overview of main mass fragments, TPD spectra of coadsorption of monolayer ethylene and a multilayer NMA, multilayer NMA and monolayer ethylene and ethylidyne/NMA at Pt(111), TPD spectra of NMA and ethylene at Pt(111), Pt 4f core level spectra, and reaction pathway following amine group dehydrogenation (PDF)
