# Supplementary material for: Role of Spectator Species for Amine-Surface Chemistry: Reactions of Amines and Alkenes on Pt(111)
Source: J Am Chem Soc. 2025 May 12;147(20):16964–71. doi: 10.1021/jacs.5c00567 (PMC12100705; doi:10.1021/jacs.5c00567)
Supplement: Supplementary file 1 [file ja5c00567_si_001.pdf]

Supporting Information

The role of spectator species for amine-surface chemistry: reactions of amines and alkenes on Pt(111)

Nils Brinkmann[*‡], Dave Austin[†‡], Bushra Ashraf[†], Duy Le[†], Talat S. Rahman[†], Katharina Al-Shamery[*]

[*] *Institute of Chemistry, Carl von Ossietzky University of Oldenburg, Carl-von-Ossietzky-Straße 9-11, 26129 Oldenburg, Germany*

[†]Department of Physics, University of Central Florida, Orlando, FL 32816, USA

‡ N.B. and D.A. contributed equally to this work.

Table of Contents:

1. Experimental Details

2. Computational Details

3. *N*-methylaniline on Pt(111)

Figure S1 Fragmentation pattern *N*-methylaniline

Figure S2 Temperature-programmed desorption spectra (TPD) of an adsorbed sub-monolayer (150 s) and a multilayer (450 s) of *N*-methylaniline at Pt(111)

Figure S3 XP spectra of an adsorbed multilayer of *N*-methylaniline (NMA) at T = 103 K at Pt(111)

4. Ethylene on Pt(111)

Figure S4 XP spectra of C1s region for an adsorbed monolayer of ethylene at T = 108 K at Pt(111)

Figure S5 Temperature-programmed desorption spectra (TPD) of ethylene adsorbed at Pt(111).

5. XPS analysis of coadsorption experiments with ethylene/NMA on Pt(111)

Figure S6 XP spectra of co-adsorption of a monolayer ethylene and a multilayer *N*-methylaniline (NMA) at T = 103 K on Pt(111) followed by heating to the specified temperatures with the N1s spectra (left) and the C1s spectra (right).

Figure S7 Temperature-programmed desorption spectra (TPD) of NMA and ethylene at Pt(111)

6. TPD analysis of coadsorption experiments

Figure S8 Overview of main mass fragments of starting compounds, decomposition products and reaction products

Figure S9: Temperature-programmed desorption spectra (TPD) of coadsorption of monolayer ethylene and a multilayer NMA, multilayer NMA and monolayer ethylene and ethylidyne/NMA at Pt(111) for the mass fragments *m/z* = 106, 92, 77, 28, 27 and 26.

Figure S10: Temperature-programmed desorption spectra (TPD) of NMA and ethylene at Pt(111).

7. Pt 4f core level spectra

Figure S11: Pt 4f core level spectra of bare Pt(111) surface and of the Pt4f surface after coadsorption of ethylidyne and NMA.

8..Reaction Pathway Following Amine Group Dehydrogenation

Figure S12: Shows the dehydrogenated NMA with ethylene. The formation of the C_3H_8N is spontaneous once the ethylene approaches the NMA.

9. References

1. Experimental Details

The presented results were measured on a self-designed ultrahigh vacuum (UHV) system with a base pressure below 10^{-10} mbar. Measurements for X-ray photoelectron spectroscopy (XPS) were carried out in a different UHV chamber than temperature-programmed desorption experiments (TPD). The UHV chambers were connected via a parking station so that the sample could be transferred between the chambers without breaking the vacuum.
The UHV chambers were equipped with a liquid nitrogen-cooled manipulator, a pin-hole dosing system for organic compounds, and an ion source with high-purity gas supply (argon, 99.999 %, Air Liquide) to the chamber through a leak valve for sputtering. The temperature of the crystal was checked by a K-type thermocouple (CHAL-005, Omega Engineering) which was spot welded to the crystal. The crystal is mounted in a home-built sample holder. The sample holder can be cooled with liquid nitrogen and can be heated by electron bombardment with a tungsten filament behind the single crystal. A further description of the sample holder system can be found elsewhere.[1]
A commercial Pt(111) single crystal (MaTeck, 10 mm diameter, 1 mm thickness) was used for surface studies. The following procedure was used to clean the Pt(111) surface. The crystal was sputtered with argon ions for 15 min at T = 298 K at $5 \cdot 10^{-5}$ mbar argon pressure and subsequently annealed for 10 min at 900 K in UHV. The cleanliness of the Pt(111) surface was checked by XPS and TPD for possibly remaining carbon residues. The Pt(111) long-range surface structure was confirmed by low-energy electron diffraction (LEED).
NMA and ethylene were dosed onto the platinum surface using a pin-hole doser, separated via a butterfly valve from the UHV chamber. For the adsorption of *N*-Methylaniline (NMA) (supplied by Thermofisher Scientific, 99 %) and ethylene (supplied by Air Liquide, 99.95%) on the platinum single crystal, the sample was cooled down with liquid nitrogen and positioned a few mm in front of the pinhole doser filled with the respective gas. For the coadsorption TPD experiments of NMA and ethylene, a pressure of 1 mbar NMA was filled into the doser, the butterfly valve to the main chamber was opened and NMA was dosed for 450 s. The dosing procedure for ethylene was analogous to NMA. A dosing pressure of 5 mbar ethylene was chosen and the main valve was opened for 360 s. To produce ethylidyne at the Pt(111) surface, ethylene was dosed first as described above and the Pt(111) single crystal was heated up to 300 K for 2 min.
For coadsorption XPS experiments, a doser pressure of $1.5 \cdot 10^{-2}$ mbar NMA was chosen and the main valve was opened for 60 s. For ethylene a pressure of $5 \cdot 10^{-1}$ mbar ethylene was filled into the doser and the main valve was opened for 120 s. Due to the different-sized pinholes between the XPS and TPD chambers, the dosing times and pressures between both chambers cannot be compared.
Temperature-programmed desorption spectra were measured with a quadrupole mass spectrometer (QMS Pfeiffer, Vacuum Prisma QMA 200) equipped with a Feulner cup (diameter of nozzle around 5 mm) and a channeltron detector. The sample was positioned a few mm in front of the Feulner cup to ensure that only molecules desorbing from the Pt(111) surface would be detected by the mass spectrometer. The sample was heated from 120 K to 900 K with a heating ramp of 2 Ks^{-1}. The mass spectra were recorded with 50 ms dwell time. To avoid systematic errors in the measured desorption peaks, the number of masses, which were measured simultaneously, was limited to 10. Spectra were divided into high mass ($m/z \geq 51$) and low mass ($m/z \leq 51$) spectra.
The UHV setup for XPS was equipped with an XPS system consisting of a Specs Phoibos 150 electron energy analyzer and a 1D-DLD detector (Surface Concept 1D-DLD64_2-150) as well as a Specs Focus 500 monochromator including a Specs XR50M X-ray source. All spectra were measured with monochromatic Al Kα radiation (1486.6 eV). Detailed XP spectra were measured with 100 ms dwell time, 0.05 eV step size, 10 eV pass energy, and 80 scans for carbon (C1s), 20 scans for nitrogen (N1s), and 2 scans for platinum (Pt4f). All spectra were referenced to bulk Pt4f = 71.1 eV. The signals were fitted with a Shirley background and Gaussian-Lorentzian curves with a Gaussian-Lorentzian ratio of GL(30) for C1s and N1s. The Pt4f signal was fitted with a Gaussian-Lorentzian ratio of GL(80) and a tailing was added to the Gaussian-Lorentzian curves for metallic platinum signals. Temperature-programmed XP-spectra were measured by applying a small heating ramp of 0.25 Ks^{-1}. TP-XP spectra were measured with 0.125 ms dwell time, 0.1 eV step size, 30 eV pass energy and one scan at the elevated temperature.

2. Computational Details

All DFT calculations were performed using the Quantum ESPRESSO (QE) software package,[2] In QE, the wave function is approximated with a plane wave basis set, with the kinetic energy cut-off set to 50 Ry and the kinetic energy cut-off for the charge density set to 500 Ry. The generalized gradient approximation proposed by Perdew-

Burke-Ernzerhof (GGA-PBE) exchange-correlation functional was utilized to describe the electronic exchange and correlation effects.[3] Furthermore, van der Waals interactions were accounted through Grimme's DFT-D3 correction.[4] Structural optimization of the atomic positions and lattice parameters were relaxed until the forces on each atom were less than the specified force threshold of 0.0001 eV/Å. The energy convergence criterion of 0.0001 eV was employed for each SCF step. This criterion ensures that the system's total energy has reached a sufficiently accurate representation of the system's electronic structure. The Brillouin zone integration was performed using a Monkhorst-Pack point mesh.[5] For computational feasibility, given that there were 63 atoms in the supercell, we sampled the Brillouin zone with a k-point mesh of 5x5x1 for all structural optimizations. To calculate the electronic density of states (DOS), a Gaussian smearing width of 0.05 eV was applied to the electronic energy levels.

3. *N*-methylaniline on Pt(111)

For the identification of mass fragments of *N*-methylaniline and its decomposition products TPD spectra were recorded according to possible mass fragments of NMA which were identified by references from the NIST database[6] and literature.[7] Figure S1 shows possible main mass fragments of NMA according to the NIST database.

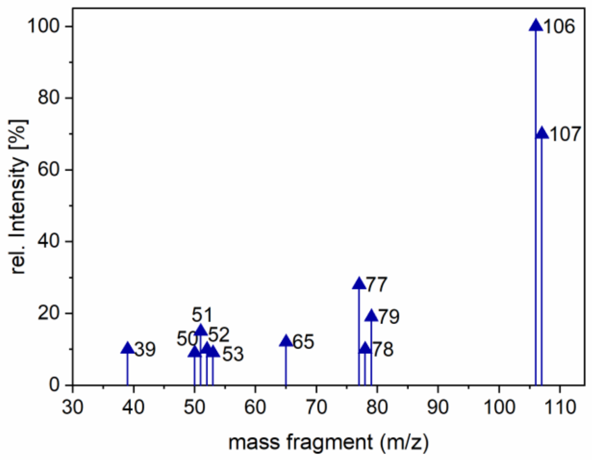

Figure S1: Main mass fragments of NMA according to the NIST database.[6] Mass fragments with less than 10 % relative intensity were not considered. Only selected *m/z* fragments were shown for better clarity. The graphic was adapted with permission, for full fragmentation pattern, we refer to the NIST database.[6]

For coverage-dependent TPD spectra, mass fragments *m/z* = 106, 77, 65, 52, 51 and 39 were selected to identify NMA desorption, besides the mass fragment *m/z* = 92 was also measured. To check for decomposition to methylamine, nitrogen, hydrogen cyanide, ammonia, methane or hydrogen, the mass fragments *m/z* = 30, 28, 27, 17, 15 and 2 were also checked. Figure S2 shows coverage-dependent TPD spectra of selected mass fragments *m/z* = 106 [$C_7H_8N^+$], 92 [$C_6H_5NH^+$], 77 [$C_6H_5^+$], 30 [CH_3NH^+], 27 [HCN^+] and 2 [H_2^+]. The high-mass spectra (*m/z* ≥ 51) were measured separately from the low-mass fragments (*m/z* ≤ 51) to avoid temperature shifts between all mass fragments. Small shifts of 2 K between the spectra are within the measurement precision and can occur due to manual opening and closing of the pinhole valve with related coverage fluctuations. The mass fragments of ammonia (*m/z* = 17) and methane (*m/z* = 15) were checked but not observed.

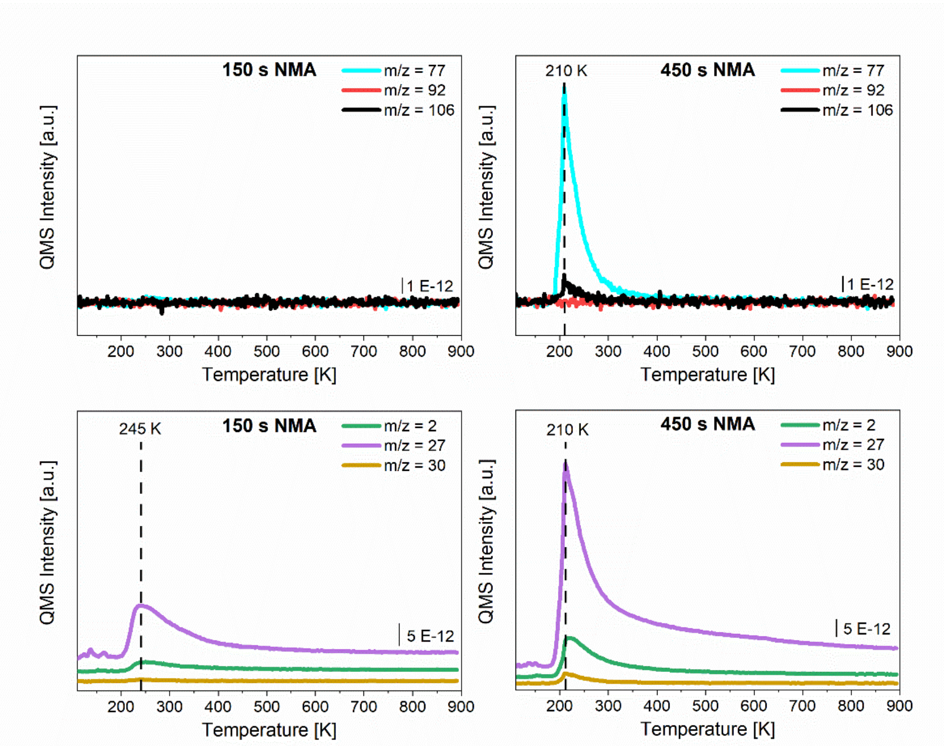

Figure S2: Temperature-programmed desorption spectra (TPD) of an adsorbed sub-monolayer (150 s) and a multilayer (450 s) of *N*-methylaniline at Pt(111) separated in high mass fragments *m/z* = 106, 92, 78 (top) and low mass fragments *m/z* = 30, 27, 2 (bottom). 1 mbar *N*-methylaniline was dosed for 450 s through a pinhole doser while the crystal was cooled to T ≤ 110 K. High-mass fragments were recorded separately from low-mass fragments.

The sub monolayer spectra of NMA do not show any desorption peaks for high-mass fragments, but the lower-mass fragments 27 [HCN^+] and 2 [H_2^+] show a desorption feature at T = 245 K, indicating that NMA decomposes on the surface, as no molecular desorption could be observed. When the dosing time is increased to 450 s, a desorption peak at T = 210 K with a shoulder at T = 240 K is observed for the higher mass fragments. A further increase in dosing time increases the low-temperature desorption feature at T = 210 K for all mass fragments (high and low), which do not reach saturation. That is why the low-temperature feature can be attributed to the multilayer desorption of NMA. The results are in good accordance with aniline desorption at Pt(111). Aniline monolayer desorption was observed at T = 240 K and multilayer desorption at T = 200 K.[8] The TPD results show that NMA dissociates at sub-monolayer coverages, while NMA remains intact after saturation of the Pt(111) surface. Multilayer adsorption of NMA was chosen for coadsorption experiments as NMA needs to be intact for further surface reactions with ethylene.

Figure S3 shows XP spectra of an adsorbed multilayer of NMA which was heated to 160 K

The study of the adsorption and surface chemistry of NMA was already the subject of a previous study.[9] Therefore, the XP spectra are briefly summarized so that this paper can focus on the changes that occurred by the coadsorption of NMA with ethylene or ethylidyne.

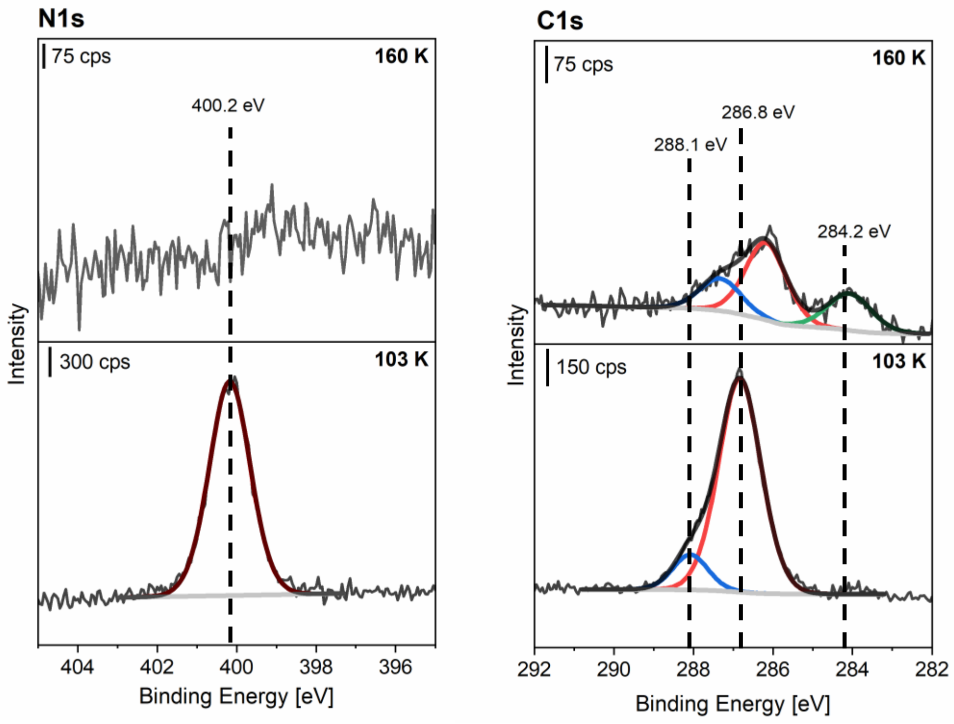

Figure S3: XP spectra of an adsorbed multilayer of *N*-methylaniline (NMA) at T = 103 K at Pt(111) followed by heating to T = 160 K with the N1s spectra (left) and the C1s spectra (right). The Pt(111) single crystal was heated for 2 min and cooled down afterward.

After exposure of the platinum surface with NMA, one intense amine signal was observed at 400.2 eV in the N1s spectrum, corresponding to the amine group of NMA.[10–13] Phenyl ring and methyl group of NMA lead to two signals at 286.8 eV and 288.1 eV with an appropriate intensity ratio of 6:1. The amine moiety in the N1s spectrum has almost completely disappeared after heating to T = 160 K, while the C1s features were down-shifted by 0.6 and 0.8 eV and an additional species at 284.2 eV appeared, indicating that thermal changes by dehydrogenation or dissociation occurred. Further heating up to 300 K (not shown) did not lead to any changes. The concrete assignment of the C1s peaks is difficult, but the peak at 284.2 eV is likely due to dehydrogenation and coking leading to C_xH_y fragments, while the carbon signals above 285 eV may be attributed to the remaining phenyl ring fragment.[14–16]

The values obtained for NMA at T = 103 K adsorbed at Pt(111) for the C1s species are up-shifted by 1.6 to 1.9 eV compared to literature results of NMA at Pt(111) at T = 300 K.[17] The reason for the strong up-shift was investigated in a previous work and has already been discussed elsewhere.[9]

4. Ethylene on Pt(111)

The following chapter presents XP and TPD spectra of ethylene on Pt(111). Figure S4 shows the C1s spectra at elevated temperatures. TP-XP spectra were recorded before (see figure 1) to identify the temperature of interest. 300 K was chosen as ethylidyne is formed at T = 283 K.

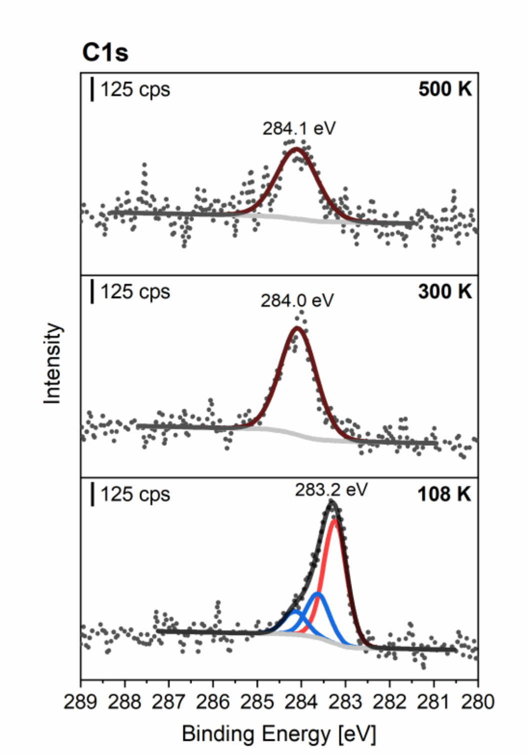

Figure S4: XP spectra of C1s region for an adsorbed monolayer of ethylene at T = 108 K at Pt(111) followed by heating to the specified temperatures. The Pt(111) single crystal was heated for 2 min to the elevated temperatures and cooled down afterward.

The TP-XPS has shown a broad C1s signal (figure 1). This observation is also confirmed by the detailed C1s spectra. After the adsorption of ethylene at Pt(111), the detailed C1s spectrum exhibits an asymmetric main peak at 283.2 eV. The shoulder at higher binding energies can be fitted with two peaks at 283.6 and 284.0 eV. The peak position and peak shape for ethylene are in good accordance with the literature.[15,18] Ethylene adsorbs molecularly through a di-σ-state parallel to the surface.[19,20] This adsorption geometry should cause only one C1s signal as both carbon atoms would be equivalent. The presence of the additional peaks can be explained by the excitation of the C-H and C-C bonds by X-ray radiation. This effect was already seen for methane and ethylene on metal surfaces before.[15,21,22] Vibrational excitation of ethylene leads to a specific binding energy separation of 0.4 eV and an intensity ratio of 0.36 between the ethylene main peak at 283.2 eV and the first excited peak at283.6 eV in the spectrum which is in good accordance with the literature.[15]
The existence of the second excited peak was also observed in synchrotron measurements before, but in contrast to the literature, the second excited peak shows a higher intensity here. This is possibly due to the different X-ray energy used in the synchrotron (380 eV) and this experiment (1486.6 eV), allowing a stronger excitation of the C-C bond. The vibrational excitation of the C-C bonds contributes to the peak shape, but cannot be resolved in XPS.[15]
Furthermore, also radiation-induced damage by forming CH fragments can contribute to the peak shape, as this

effect was observed for methane at Pt(111).[21] The possibility of radiation damage cannot be excluded here and is increasing due to the longer measurement time and higher photon energy compared to synchrotron measurements. When heated up to 300 K, the literature-known ethylidyne species is formed,[16,23] as can be seen from the up-shift of the signal to 284.0 eV.[15,18] Further heating to T = 500 K (see figure S4) only results in a small decrease in the intensity and broadening of the signal indicating a further decomposition by dehydrogenation of the ethylidyne to carbon residues (C_xH_y).[23] The TP-XPS in figure 1 shows that the coking starts at T = 400 K. The carbon residues remain at the surface, as was already proven by TP-XPS before.
Figure S5 shows the temperature-programmed desorption spectra (TPD) of a monolayer of ethylene adsorbed at Pt(111). Ethylene has the main mass fragments m/z = 28 [$CO^+/C_2H_4^+$], 27 [$C_2H_3^+$] and 26 [$C_2H_2^+$]. Simultaneous desorption peaks of these mass fragments are due to ethylene desorption. Figure S5 shows all mass fragments, which were detected for the adsorption of ethylene at Pt(111).

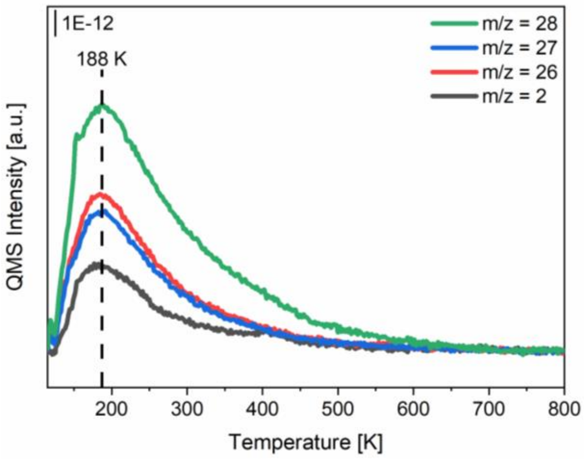

Figure S5: Temperature-programmed desorption spectra (TPD) of ethylene adsorbed at Pt(111). 5 mbar Ethylene was dosed for 360 s through a pinhole doser while the crystal was cooled to T ≤ 110 K.

The mass fragments m/z = 12, 15, 16 and 30 were measured, but not detected. Therefore, the formation of methane or ethane can be excluded here. The TPD spectra show a broad ethylene desorption peak at T = 188 K for the mass fragments 28 [$CO^+/C_2H_4^+$], 27 [$C_2H_3^+$] and 26 [$C_2H_2^+$]. Besides ethylene desorption, also hydrogen desorption m/z = 2 is observed. Besides the main peak at T = 188 K, an additional small shoulder at T = 405 K can be observed, indicating a further dehydrogenation process. The observed desorption temperature of T = 188 K is in contrast to literature values of T = 300 K observed for Pt(111).[20,24] However, similarly low desorption temperatures of 183 K could be observed on tin-alloyed platinum single crystals.[25,26] One possible reason is that the heating rate (2 Ks^{-1}) was by a factor of five lower compared to the literature. In general, the heating rate influences the temperature for the maximum of desorption. A down-shift can be observed by decreasing the heating rate.[27,28] However, the different heating rates cannot explain the downshift alone. The influence of coadsorbed hydrogen from the background pressure of the chamber cannot be excluded here. It was observed for platinum and tin-platinum alloys that coadsorbed hydrogen leads to a weaker interaction of the ethylene with the platinum surface also leading to a broadening of the desorption peak.[29,30] A broad desorption peak at T = 200 K was observed for Pt(111), which is in good agreement with the results presented here.[29] Nevertheless, the broad desorption peak observed in this work may also hint to a low pumping speed or interactions of the ethylene with the steel wall of the Feulner cup.

5. XPS analysis for coadsorption of ethylene and NMA on Pt(111)

In front of the co-adsorption studies, the adsorption behavior and surface chemistry of NMA and ethylene were investigated individually to establish the benchmarks for co-adsorption and have been discussed before. This chapter only focuses on the co-adsorption results, starting with the X-ray photoelectron (XP) spectra for the co-adsorption of NMA on an ethylene-pre-covered surface (figure S6), followed by the co-adsorption in reverse order (figure S7).

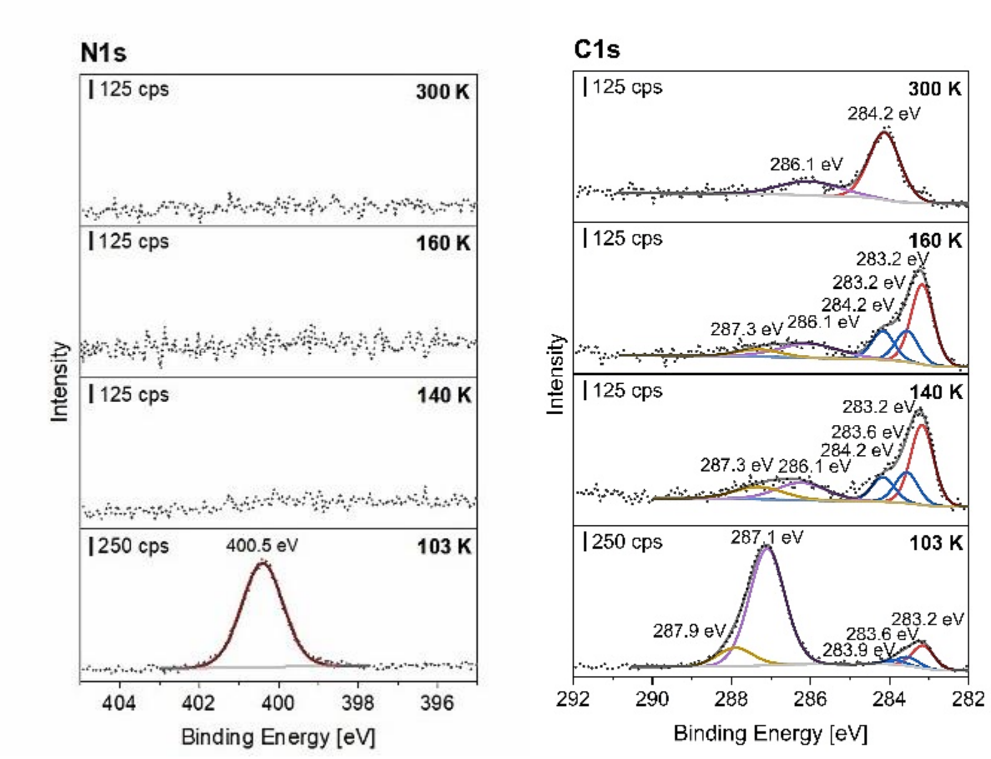

Figure S6: XP spectra of co-adsorption of a monolayer ethylene and a multilayer *N*-methylaniline (NMA) at T = 103 K on Pt(111) followed by heating to the specified temperatures with the N1s spectra (left) and the C1s spectra (right). First ethylene was dosed onto the surface and NMA was dosed subsequently onto the ethylene pre-covered surface. The Pt(111) single crystal was heated for 2 min to the elevated temperatures and cooled down before XP spectra were collected at T≤110 K.

Figure S6 shows the XP spectra, starting with ethylene adsorption, followed by NMA adsorption on the ethylene pre-covered surface and the decomposition during heating up to 300 K. After co-adsorption, the N1s spectrum exhibits a broad amine signal at 400.5 eV, while the C1s detailed spectrum shows two signals for NMA at 287.1 and 287.9 eV and one signal for ethylene at 283.2 eV with its additional shoulders related to the X-ray excitation of the C-H and C-C bonds.[15,21] Vibrational excitation of ethylene leads to a specific binding energy separation of 0.4 eV and an intensity ratio of 0.36 between the ethylene main peak at 283.2 eV and the first excited peak at 283.6 eV in the spectrum which is in good accordance with the literature.[15] The second excited peak is also due to the excitation of the C-H bonds. [13,20,21]
The area ratio between the signals at 287.1 and 287.9 eV is about 6:1 and can be assigned to the methyl group and phenyl ring of NMA and is in good accordance to experiments of NMA on Pt(111) reported before.[9]
The co-adsorption spectra do not show significant differences compared to the separate adsorption of NMA (see Figure S3) and ethylene (see Figure S4) at Pt(111) at liquid nitrogen temperature. Slight differences in the binding energy of ± 0.2 eV are within the measurement precision.
Thermally induced changes and the formation of a new decomposition surface species are visible when heated up to 140 K. While the amine signal has vanished in the N1s spectrum, both NMA C1s signals are still present, but strongly decreased in intensity and downshifted to 286.1 and 287.3 eV, similar to the results for NMA only at Pt(111).

The thermal changes in the XP spectra can be attributed to the formation of a new surface species by dehydrogenation or dissociation of the phenyl ring. The ethylene signal at 283.2 eV does not change in intensity or binding energy, except for the shoulder at 283.9 eV which is upshifted to 284.2 eV and increased in intensity. Results from NMA at Pt(111) have shown the evolution of a shoulder at 284,2 eV by the formation of dehydrogenated C_xH_y fragments. The above-described up-shift of the second excited ethylene peak signal results from the overlap of a dehydrogenated C_xH_y species of NMA at 284.2 eV and the X-ray-induced excitation of vibrational modes.[9,15,21] A concrete assignment of this fragment is difficult, but the binding energy of the signal corresponds to sp^3 hybridized carbon.[16] The development of a shoulder at lower binding energies by heating up was also observed for the dehydrogenation of the methyl group of acetophenone, however as the C1s signals of the phenyl ring strongly change upon heating, a competing dehydrogenation of the ring cannot be excluded here.[31] The downshift of the NMA signals and the lack of the N1s signal hint to the start of a decomposition of the ring.

The signal at 287.3 eV disappears by further heating up to T = 300 K while the signal at 286.1 eV does not change, indicating that the methyl group of NMA has decomposed while a dehydrogenated fragment of the phenyl ring remains at the surface.

To summarize, the coadsorption on the ethylene-pre-covered surface shows a similar surface chemistry for NMA as compared to that from NMA only, but with a stronger decrease in intensity and the disappearance of the signal at 287.3 eV during heating. The upshift of the C1s signal from 283.2 eV to 284.2 eV in Fig. S6 indicates the conversion of ethylene to ethylidyne.

The co-adsorption experiment was performed in reverse order to elucidate whether the sequence of adsorption influences the surface chemistry of both components. Figure S7 shows the XP spectra for the adsorption of ethylene at an NMA-pre-covered surface at Pt(111).

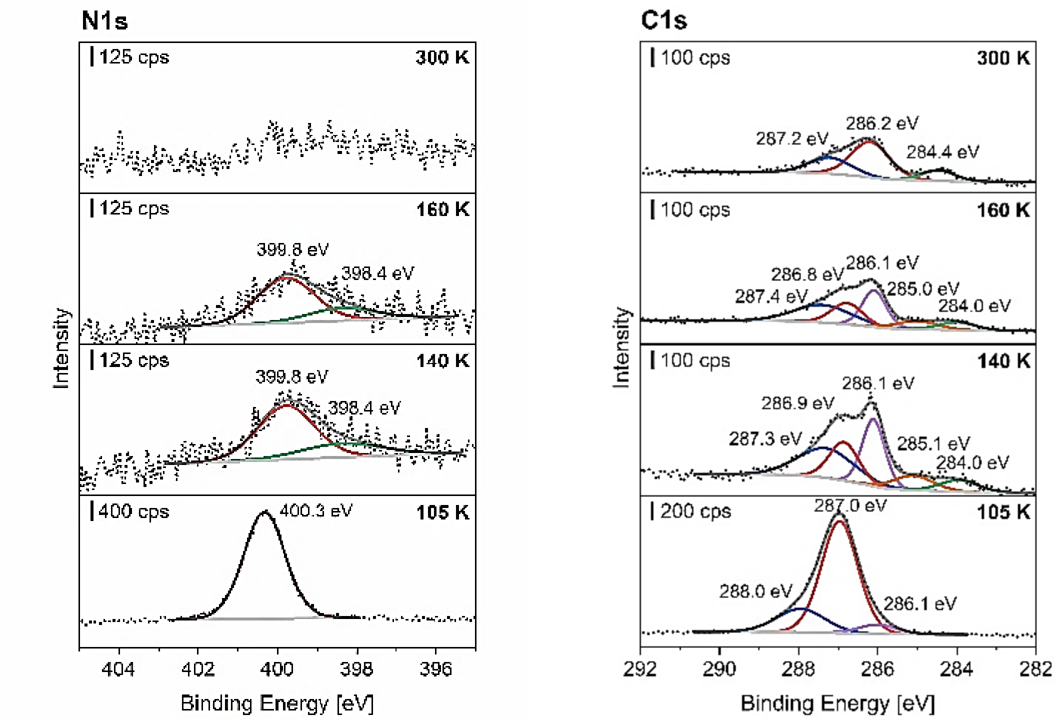

Figure S7: XP spectra of co-adsorption of a multilayer *N*-methylaniline (NMA) and a monolayer ethylene at T = 105 K on Pt(111) followed by heating to the specified temperatures with the N1s spectra (left) and the C1s spectra (right). First NMA was dosed onto the surface and then ethylene onto the NMA pre-covered surface. The Pt(111) single crystal was heated for 2 min to the elevated temperatures and cooled down before XP spectra were collected at T≤110 K.

The co-adsorption spectra of NMA and ethylene show a broad amine signal at 400.3 eV in the N1s spectrum and three signals at 288.0, 287.0 and 286.1 eV in the C1s spectrum after adsorption at the Pt(111) surface. No C1s

signal at 283.2 eV is observed in contrast to the ethylene-pre-covered surface before, indicating that the additional species at 286.1 eV is possibly a reaction product of ethylene with NMA or one of its decomposition products.
Significant changes in the spectra occur during heating up to T = 140 K. The amine signal in the N1s spectrum strongly decreases in intensity and is downshifted to 399.8 eV while an additional shoulder develops at 398.4 eV. Both signals are present up to T = 160 K, but disappear during further heating up to 300 K.
In the C1s spectrum, two new shoulders develop by heating up to T = 140 K at 285.1 and 284.0 eV. The signals at 288.0 eV and 287.0 eV decrease in intensity but the signal at 286.1 eV increases. When heating up to 160 K, the intensity of all signals decrease, which indicates a desorption of the species from the platinum surface. On further heating to 300 K, the species at 285.0 and 286.1 eV have vanished in the C1s spectrum, while three signals at 284.4, 286.2 and 287.2 eV appear.
The species at 286.1 eV which is already formed after the co-adsorption of ethylene is in the region of a newly formed C-N bond and fits to a formation of methylamine.[32–34] In the N1s spectrum, it is not possible to differentiate between the amines, therefore the amine signal at 400.3 eV results from the superposition of NMA and the newly formed amine. For further identification of this species, TPD spectra were measured, and a concrete assignment of this species is given using TPD spectra later. The signals at 285.0 and 398.4 eV can be assigned to hydrocyanic acid.[32,33]
The co-adsorption spectra show some differences compared to the separate adsorption of NMA and ethylene at the Pt surface (see Figures S3 and S4). While the NMA signals do not show a difference compared to NMA only after adsorption, the up-shift of the ethylene signal to 286.1 eV by possible C-N bond formation is noticeable. Furthermore, the formation of HCN has not been observed in the XP-spectra before. Further differences appear during decomposition when heating. The amine is present up to T = 160 K in coadsorption experiments indicating an increased stabilization of NMA by co-adsorption with ethylene. After the desorption of HCN and the additional species at 286.1 eV, the NMA decomposition does not show a difference to NMA only at Pt(111), so the same decomposition species on the surface can be assumed.

6. TPD analysis for coadsorption experiments

The XP spectra of ethylene at Pt(111) (see Figure S4) exhibit a chemical transformation step from ethylene to ethylidyne species.[15] The TPD spectra in Figure S5 show that even at lower temperatures (e.g. T = 188 K) molecular desorption of a part of ethylene already takes place with a broad desorption peak in the mass fragments m/z = 28 [$CO^+/C_2H_4^+$], 27 [$C_2H_3^+$], 26 [$C_2H_2^+$] and 2 [H_2^+]. Ethylidyne represents an interesting surface species, which can probably act as a spectator or influence the surface chemistry of NMA by enrichment of the platinum surface with hydrogen. Therefore, adsorption of NMA was also carried out in the presence of this species at the surface.
For the identification of relevant mass fragments the NIST database was used.[6] An overview of relevant mass fragments of NMA, ethylene, decomposition and reaction products is given in Figure S8.

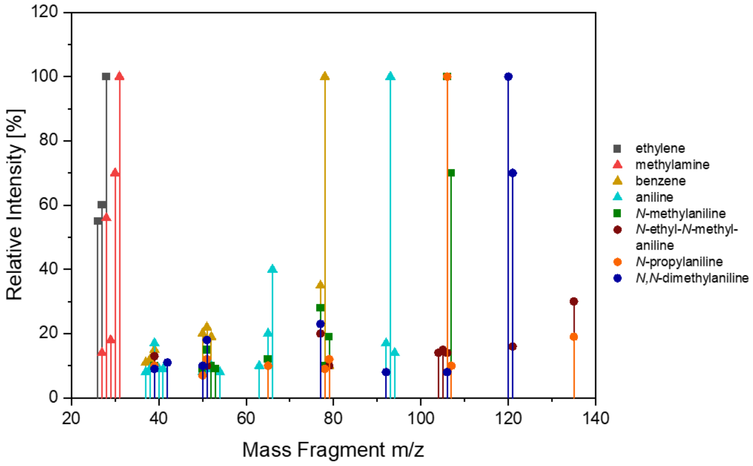

Figure S8: Overview of main mass fragments of starting compounds (▪), decomposition products (▲) and reaction products (●). Fragmentation pattern was extracted from the NIST database. Only selected

m/z fragments were shown for better clarity. The graphic was adapted with permission, for full fragmentation pattern, we refer to the NIST database.[6]

NMA exhibits the mass fragments *m/z* = 106 [$C_7H_8N^+$], 78 [$C_6H_6^+$], 77 [$C_6H_5^+$], 52 [C_2N_2], 30 [CH_3NH^+] 28 [CO^+/N_2^+] 27 [HCN^+] and 2 [H_2^+]. Some mass fragments overlap with ethylene as exhibited by the mass fragments *m/z* = 28 [$CO^+/C_2H_4^+$], 27 [$HCN^+/C_2H_3^+$] and 26 [$CN^+/C_2H_2^+$]. For identification of reaction products by hydroamination or hydroaminoalkylation, especially the higher mass fragments are of importance. *N*-ethyl-*N*-methylaniline (NEMA) and *N*-propylaniline (NPA) can be distinguished by the mass fragments *m/z* = 135 [$C_9H_{13}N^+$], 121[$C_8H_{11}N$] and 120 [$C_8H_{10}N^+$]. While both products exhibit the mass fragment *m/z* = 135[$C_9H_{13}N^+$], only NEMA shows mass fragments m/z = 121 [$C_8H_{11}N$] and 120 [$C_8H_{10}N^+$].
TPD spectra were recorded for the co-adsorption experiments, starting with the high-mass fragments in figure S9 (*m/z* = 106 [$C_7H_8N^+$], 92 [$C_6H_5NH^+$], 77 [$C_6H_5^+$]). The lower-mass fragments were recorded separately and will be discussed further below.
The spectra (**A/D**) in figure S9 present the spectra of ethylene on the NMA-pre-covered Pt surface. The TPD spectra (**B/E**) are related to NMA adsorption on the ethylene-pre-covered Pt surface and the TPD spectra (**C/F**) show the NMA adsorption on the ethylidyne-pre-covered Pt surface. Ethylidyne was formed by dosing ethylene first at liquid nitrogen temperature and subsequent heating to T = 298 K keeping the temperature for 2 min.

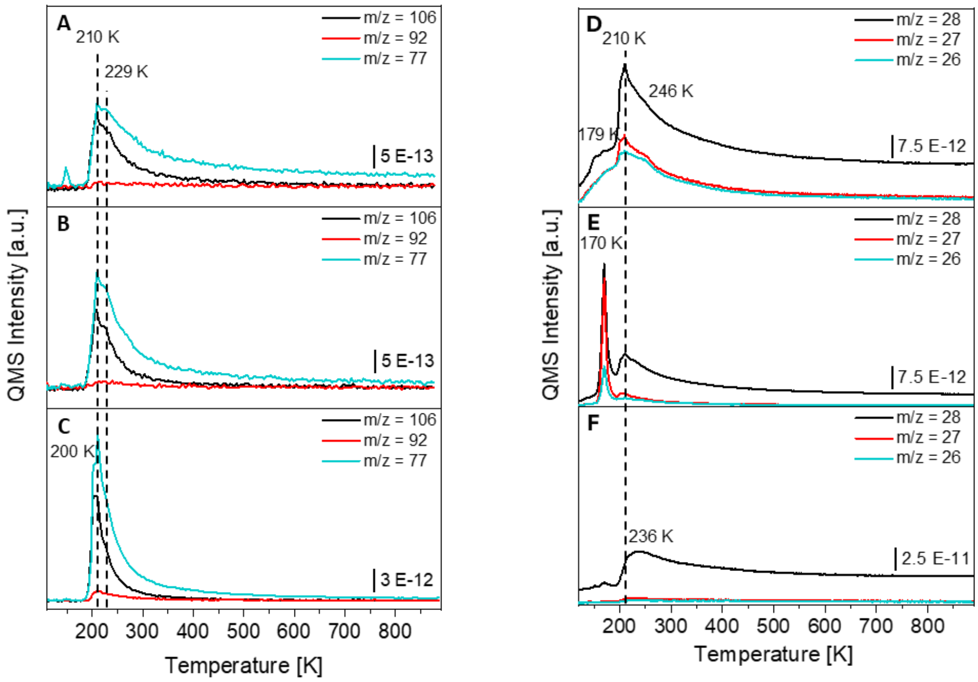

Figure S9: Temperature-programmed desorption spectra (TPD) of coadsorption of monolayer ethylene and a multilayer NMA (**A/D**), multilayer NMA and monolayer ethylene (**B/E**) and ethylidyne/NMA (**C/F**) at Pt(111). The high mass fragments *m/z* = 106, 92 and 77 are shown left, and the low mass fragments *m/z* = 28, 27 and 26 are shown right. The surface was dosed first with the component named first and the second component was then dosed on the pre-covered surface while the crystal was cooled to T ≤ 110 K. Ethylidyne was formed by heating the crystal to 298 K for 2 min after the adsorption of a monolayer ethylene. The ethylidyne pre-covered surface was then exposed to a multilayer NMA at liquid nitrogen temperature.

All TPD spectra seem similar at first glance independent of the order of dosing. However, there is an important difference in the case of the ethylidyne pre-covered platinum surface (**C**). First, all spectra have a desorption peak at T = 210 K with a high-temperature shoulder at T = 229 K for the mass fragments *m/z* = 106 [$C_7H_8N^+$], and 77 [$C_6H_5^+$]. These desorption peaks are in good accordance with NMA adsorption at Pt(111) (see figure S2). The low-temperature feature at T = 210 K can be assigned to multilayer desorption, while the high-temperature feature at T = 229 K is due to the desorption of the NMA monolayer.[8] Spectrum (**A**) shows an artifact at T = 150 K only for this particular mass, which is not further discussed here.

However, in the presence of ethylidyne on the platinum surface, TPD spectrum (**C**) exhibits an additional shoulder at T = 200 K (see also figure 4). The spectrum also shows a small desorption peak for mass fragment *m/z* = 92 [$C_6H_5NH^+$]. Furthermore, it is noteworthy that the NMA desorption peaks exhibit a six-time enhanced intensity, indicating that a higher amount of NMA is desorbing intact from the surface. The presence of ethylidyne on the surface seems to suppress the decomposition of the NMA. This is confirmed by the low mass fragment TPD spectrum (**F**). In the presence of ethylidyne (**F**), the TPD spectrum shows only for mass fragment *m/z* = 28 [CO^+/$C_2H_4^+$] a broad desorption peak at T = 236 K. In contrast to NMA only or in coadsorption with ethylene (**D**), (**E**) no desorption can be observed for the fragment's *m/z* = 27 [HCN^+/$C_2H_3^+$] and 26 [$C_2H_2^+$].
The occurrence of the mass fragment *m/z* = 92 [$C_6H_5NH^+$] is due to the formation of *N,N*-dimethylaniline (see Figure 4), as additional mass fragments *m/z* = 121 [$C_8H_{11}N$] and 120 [$C_8H_{10}N^+$] have been measured which is discussed in the main article.
Spectrum (**A**) for ethylene on an NMA-pre-covered Pt surface shows extensive desorption to much higher temperatures for the benzene fragment *m/z* = 77 [$C_6H_5^+$]. As apparent from XP spectra in Figure S6, there is an ongoing decomposition of the remaining aromatic ring fragment up to temperatures to 300 K. This ongoing decomposition of the ring may cause a delayed desorption of the fragment *m/z* = 77 [$C_6H_5^+$]. The hydrogen which is available on the surface by the decomposition of the NMA molecule, could re-hydrogenate the dehydrogenated aromatic ring species which would rearrange to form benzene (C_6H_6).
After discussing the high-mass fragments, the low-mass fragment TPD spectra are discussed in the following. The TPD spectrum (**D**) for NMA on an ethylene-pre-covered Pt surface shows a broad shoulder that reaches a plateau at T = 179 K, followed by an intense desorption peak at T = 210 K, which also exhibits another shoulder at T = 246 K. When changing the sequence of adsorption, the TPD spectrum (**E**) changes significantly. The TPD spectrum for the NMA-pre-covered surface shows two main desorption features, but the desorption peak at T = 170 K is the most intense and significantly increased compared to spectrum (**D**).
The desorption peak at T = 210 K in spectrum (**D**/**E**) can be assigned to the desorption of NMA. It is noticeable that the desorption signal is significantly broader compared to the NMA only, indicating an overlapping desorption peak of ethylene at similar temperatures. Ethylene desorption was observed without coadsorption of NMA at T = 188 K. Due to the small temperature difference between NMA multilayer desorption and ethylene desorption, both desorption peaks overlap, leading to a broadening of the NMA desorption peak.
One could assume that the desorption peak at T = 170 K in the TPD spectrum (**E**) could be assigned to ethylene desorption, but two facts contradict this point. On the one hand, the ratio of the mass fragments *m/z* = 27 [HCN^+/$C_2H_3^+$] and 26 [CN^+/$C_2H_2^+$] does not match here and on the other hand the intensities of these desorption peaks are too high for ethylene desorption. The ratio between *m/z* = 27 and 26 is approximately the same for ethylene, but for the 170 K peak the ratio is 3,5:1. Since ethylene does not show multilayer adsorption on Pt(111) at temperatures around 100 K, the ethylene desorption signal should have a much lower relative intensity than the NMA desorption signal. The ethylene desorption can be identified in spectrum (**D**) as broad shoulder with low intensity at T = 179 K because the relative intensity and absolute intensity fit to the ethylene desorption observed in test experiments before (see Figure S5).
The desorption feature at T = 170 K is due to the formation of a side product by the reaction of ethylene with decomposition products of NMA, as besides the ethylene fragments *m/z* = 28, 27 and 26, also the further fragments *m/z* = 59 [$C_3H_9N^+$], 31[$CH_3NH_2^+$], 15 [CH_3^+] and 12 [C^+] which do not belong to ethylene desorption (see Fig. S10) were measured. These mass fragments could be due to the formation of ethyl methyl amine. The large mass fragments *m/z* ≥ 51 do not show any desorption feature at T = 170 K. The desorption peak can be assigned to one or more dissociation products of NMA. The ratio between *m/z* = 27 [HCN^+/$C_2H_3^+$] and 26 [CN^+/$C_2H_2^+$] fits to the fragmentation pattern of HCN, and mass fragments *m/z* = 31 [$CH_3NH_2^+$] and 15 [CH_3^+] indicate a desorption of methylamine. The TPD spectrum indicates that the presence of ethylene favors the dissociation of the NMA, however this dissociation leads to the formation of several products via competing reaction pathways.

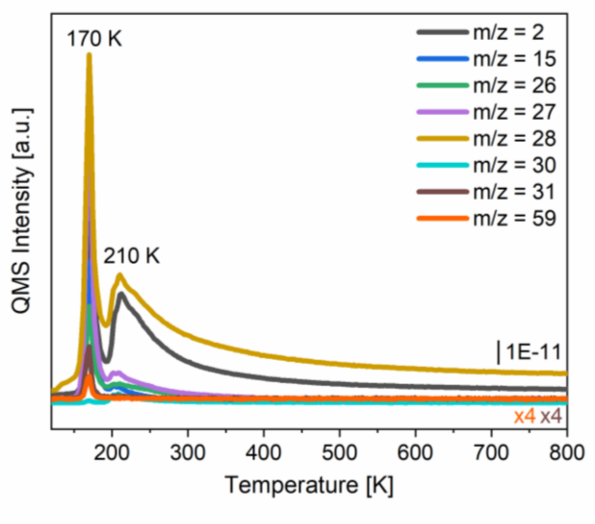

Figure S10: Temperature-programmed desorption spectra (TPD) of NMA and ethylene at Pt(111). The intensity of the mass fragments *m/z* = 59 and 31 was multiplied with a factor of four for a better visibility.

Besides the intense peaks for the mass fragments *m/z* = 28, 27, 26, 15 and 2, the 170 K peak exhibits also some less intense desorption features for the mass fragments *m/z* = 59 [$C_3H_9N^+$], and 31 [$CH_3NH_2^+$]. Due to the high intensity, the intensity ratio between *m/z* = 27 [$HCN^+/C_2H_3^+$] and 26 [$CN^+/C_2H_2^+$] of 3,5 to 1 and the presence of additional mass fragments, this peak cannot be caused by ethylene desorption alone. The intensity ratio of 3,5 to 1 hints to the fact that HCN is desorbing from the surface, but the presence of *m/z* = 59 [$C_3H_9N^+$], and 31 [$CH_3NH_2^+$] indicates that also a small amount of methylamine and ethyl methyl amine is formed.

7. Pt 4f core level spectra

Figure S11 shows the Pt 4f core level spectra. The red curve show the bare Pt 4f core level spectrum, while the blue curve fits show the Pt 4f core level spectrum of the coadsorption of ethylidyne and NMA at liquid nitrogen temperature.

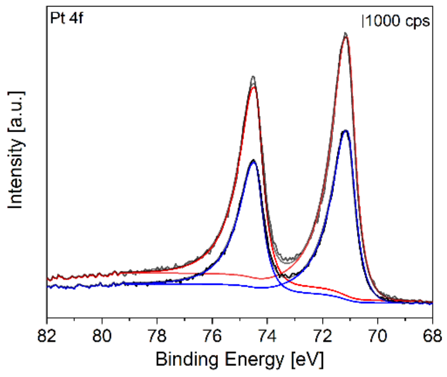

Figure S11: Pt 4f core level spectra of bare Pt(111) surface (red curve fits) and of the Pt4f surface after coadsorption of ethylidyne and NMA at liquid nitrogen temperature.

The Pt 4f signals were referenced to bulk Pt4f = 71.1 eV. Pt 4f core-level spectra do not show a strong decrease in intensity compared to the clean Pt(111) surface. This indicates that a significant portion of the Pt surface remains exposed and is not covered by a thick adlayer. Therefore, our experimental conditions are consistent with the theoretical model in which reactions occur on Pt(111).

8. Reaction Pathway Following Amine Group Dehydrogenation

DFT calculations were performed that underscore the importance of N-H activation in forming ethyl methyl amine precursor C_3H_6N. Following the dehydrogenation of the amine group, which increases the nitrogen atom's reactivity, ethylene can readily interact with the amine. This interaction is followed by the activation of the C–N bond between the amine and phenyl group, a crucial step in forming ethyl methyl amine, as illustrated in Figure S12. The necessity of dehydrogenating the *N*-methylaniline (NMA) molecule arises from its role in enhancing nitrogen reactivity (see figure 4), thereby enabling more effective interaction with ethylene

When ethylene approaches the dehydrogenated NMA, a spontaneous reaction occurs, leading to the formation of the C_3H_6N precursor. This process is energetically favorable, requiring only a modest energy increase of 0.46 eV. The formation of the C_3H_6N molecule is direct and underscores the significance of the initial dehydrogenation step. By increasing the nitrogen atom's reactivity, dehydrogenation facilitates subsequent transformations, ultimately contributing to the efficient synthesis of complex organic molecules.

Overall, this mechanism highlights the essential role of dehydrogenation in modulating chemical reactivity and emphasizes the intricate relationship between molecular structure and reaction dynamics.

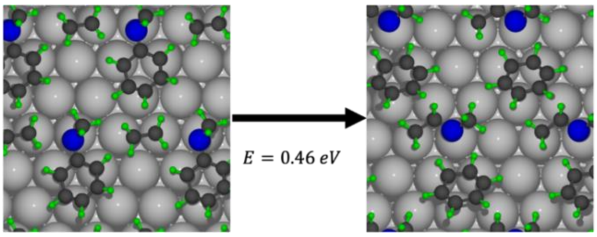

Figure S12: Shows the dehydrogenated NMA with ethylene. The formation of the C_3H_6N is spontaneous once the ethylene approaches the NMA.

9. References

(1) Leist, U.; Winkler, A.; Büssow, J.; Al-Shamery, K. Mobile sample holder applying multiple heating systems with a variable heating and cooling rate. *Review of Scientific Instruments* **2003**, *74* (11), 4772–4778. DOI: 10.1063/1.1614413.

(2) Giannozzi, P.; Baroni, S.; Bonini, N.; Calandra, M.; Car, R.; Cavazzoni, C.; Ceresoli, D.; Chiarotti, G. L.; Cococcioni, M.; Dabo, I.; Dal Corso, A.; Gironcoli, S. de; Fabris, S.; Fratesi, G.; Gebauer, R.; Gerstmann, U.; Gougoussis, C.; Kokalj, A.; Lazzeri, M.; Martin-Samos, L.; Marzari, N.; Mauri, F.; Mazzarello, R.; Paolini, S.; Pasquarello, A.; Paulatto, L.; Sbraccia, C.; Scandolo, S.; Sclauzero, G.; Seitsonen, A. P.; Smogunov, A.; Umari, P.; Wentzcovitch, R. M. QUANTUM ESPRESSO: a modular and open-source software project for quantum simulations of materials. *Journal of physics. Condensed matter : an Institute of Physics journal* **2009**, *21* (39), 395502. DOI: 10.1088/0953-8984/21/39/395502. Published Online: Sep. 1, 2009.

(3) Perdew, J. P.; Burke, K.; Ernzerhof, M. Generalized Gradient Approximation Made Simple. *Physical review letters* **1996**, *77* (18), 3865–3868. DOI: 10.1103/PhysRevLett.77.3865.

(4) Grimme, S. Accurate description of van der Waals complexes by density functional theory including empirical corrections. *Journal of computational chemistry* **2004**, *25* (12), 1463–1473. DOI: 10.1002/jcc.20078.

(5) Monkhorst, H. J.; Pack, J. D. Special points for Brillouin-zone integrations. *Phys. Rev. B* **1976**, *13* (12), 5188–5192. DOI: 10.1103/PhysRevB.13.5188.

(6) Linstrom, P. *NIST Chemistry WebBook, NIST Standard Reference Database 69*.

(7) Rinehart, K. L.; Buchholz, A. C.; van Lear, G. E. Mass Spectral fragmentation of Aniline-1-13C. *Journal of the American Chemical Society* **1968**, *90* (4), 1073–1075.

(8) Huang, S. X.; Fischer, D. A.; Gland, J. L. Correlation between the surface configurations and hydrogenolysis: Aniline on the Pt(111) surface. *Journal of Vacuum Science & Technology A: Vacuum, Surfaces, and Films* **1994**, *12* (4), 2164–2169. DOI: 10.1116/1.579107.

(9) Ashraf, B.; Brinkmann, N.; Austin, D.; Lee, D.; Al-Shamery, K.; Rahman, T. S. Unveiling coverage dependent interaction of N-methylaniline at the Pt(111) surface. *J. Phys. Chem. C* **2025**, *129*, 6196–6210. DOI: 10.1021/acs.jpcc.4c08116.

(10) Bonello, J. M.; Williams, F. J.; Lambert, R. M. Aspects of enantioselective heterogeneous catalysis: structure and reactivity of (S)-(-)-1-(1-naphthyl)ethylamine on Pt111. *Journal of the American Chemical Society* **2003**, *125* (9), 2723–2729. DOI: 10.1021/ja028436x.

(11) Siemer, M.; Tomaschun, G.; Klüner, T.; Christopher, P.; Al-Shamery, K. Insights into Spectator-Directed Catalysis: CO Adsorption on Amine-Capped Platinum Nanoparticles on

Oxide Supports. *ACS applied materials & interfaces* **2020**, *12* (24), 27765–27776. DOI: 10.1021/acsami.0c06086. Published Online: Jun. 3, 2020.

(12) Brinkmann, N.; Damps, A.; Siemer, M.; Kräuter, J.; Rößner, F.; Al-Shamery, K. Catalytic Reactions at Amine-Stabilized and Ligand-Free Platinum Nanoparticles Supported on Titania during Hydrogenation of Alkenes and Aldehydes. *Journal of visualized experiments : JoVE* **2022** (184). DOI: 10.3791/63936. Published Online: Jun. 24, 2022.

(13) Adenier, A.; Chehimi, M. M.; Gallardo, I.; Pinson, J.; Vilà, N. Electrochemical oxidation of aliphatic amines and their attachment to carbon and metal surfaces. *Langmuir : the ACS journal of surfaces and colloids* **2004**, *20* (19), 8243–8253. DOI: 10.1021/la049194c.

(14) Sundararajan, R. Photoemission studies on Pt foil implanted by carbon atoms accelerated in a Van de Graaff generator: nature of the interaction between Pt and carbon. *Applied Surface Science* **1995**, *90* (2), 165–173. DOI: 10.1016/0169-4332(95)00060-7.

(15) Fuhrmann, T.; Kinne, M.; Tränkenschuh, B.; Papp, C.; Zhu, J. F.; Denecke, R.; Steinrück, H.-P. Activated adsorption of methane on Pt(1 1 1) —an in situ XPS study. *New J. Phys.* **2005**, *7*, 107. DOI: 10.1088/1367-2630/7/1/107.

(16) Zaera, F. Formation and thermal decomposition of ethyl groups on transition metal surfaces: Ethyl iodide on Pt(111). *Surface Science* **1989**, *219* (3), 453–466. DOI: 10.1016/0039-6028(89)90521-9.

(17) Mudiyanselage, K.; Trenary, M. Adsorption and thermal decomposition of N-methylaniline on Pt(111). *Surface Science* **2009**, *603* (21), 3215–3221. DOI: 10.1016/j.susc.2009.09.005.

(18) Freyer, N.; Pirug, G.; Bonzel, H. P. C(1s) spectroscopy of hydrocarbons adsorbed on Pt(111). *Surface Science* **1983**, *126* (1-3), 487–494. DOI: 10.1016/0039-6028(83)90747-1.

(19) Steininger, H.; Ibach, H.; Lehwald, S. Surface reactions of ethylene and oxygen on Pt(111). *Surface Science* **1982**, *117* (1-3), 685–698. DOI: 10.1016/0039-6028(82)90549-0.

(20) Zaera, F. On the Mechanism for the Hydrogenation of Olefins on Transition-Metal Surfaces: The Chemistry of Ethylene on Pt(111). *Langmuir : the ACS journal of surfaces and colloids* **1996**, *12* (1), 88–94. DOI: 10.1021/la9407020.

(21) Fuhrmann, T.; Kinne, M.; Whelan, C. M.; Zhu, J. F.; Denecke, R.; Steinrück, H.-P. Vibrationally resolved in situ XPS study of activated adsorption of methane on Pt(111). *Chemical Physics Letters* **2004**, *390* (1-3), 208–213. DOI: 10.1016/j.cplett.2004.04.030.

(22) Andersen, J.N.; Beutler, A.; Sorensen, S.L.; Nyholm, R.; Setlik, B.; Heskett, D. Vibrational fine structure in the C 1s core level photoemission of chemisorbed molecules: ethylene and ethylidyne on Rh(111). *Chemical Physics Letters* **1997**, *269* (3-4), 371–377. DOI: 10.1016/S0009-2614(97)00288-1.

(23) Land, T. A.; Michely, T.; Behm, R. J.; Hemminger, J. C.; Comsa, G. Direct observation of surface reactions by scanning tunneling microscopy: Ethylene→ethylidyne→carbon

particles→graphite on Pt(111). *The Journal of Chemical Physics* **1992**, *97* (9), 6774–6783. DOI: 10.1063/1.463655.

(24) Godbey, D.; Zaera, F.; Yeates, R.; Somorjai, G. A. Hydrogenation of chemisorbed ethylene on clean, hydrogen, and ethylidyne covered platinum (111) crystal surfaces. *Surface Science* **1986**, *167* (1), 150–166. DOI: 10.1016/0039-6028(86)90791-0.

(25) Tsai, Y.-L.; Xu, C.; Koel, B. E. Chemisorption of ethylene, propylene and isobutylene on ordered Sn/Pt(111) surface alloys. *Surface Science* **1997**, *385* (1), 37–59. DOI: 10.1016/S0039-6028(97)00114-3.

(26) Zhao, H.; Koel, B. E. Reactivity of Ethyl Groups on a Sn/Pt(111) Surface Alloy. *Catal Lett* **2005**, *99* (1-2), 27–32. DOI: 10.1007/s10562-004-0772-6.

(27) Kanervo, J. M.; Reinikainen, K. M.; Krause, A.O.I. Kinetic analysis of temperature-programmed desorption. *Applied Catalysis A: General* **2004**, *258* (2), 135–144. DOI: 10.1016/j.apcata.2003.08.019.

(28) Rakić, V.; Damjanović, L. Temperature-Programmed Desorption (TPD) Methods. In *Calorimetry and Thermal Methods in Catalysis*; Auroux, A., Ed.; Springer Series in Materials Science; Springer Berlin Heidelberg, 2013; pp 131–174. DOI: 10.1007/978-3-642-11954-5_4.

(29) Berlowitz, P.; Megiris, C.; Butt, J. B.; Kung, H. H. Temperature-programmed desorption study of ethylene on a clean, a hydrogen-covered, and an oxygen-covered platinum(111) surface. *Langmuir : the ACS journal of surfaces and colloids* **1985**, *1* (2), 206–212. DOI: 10.1021/la00062a005.

(30) Zhao, H.; Koel, B. E. Influence of coadsorbed hydrogen on ethylene adsorption and reaction on a (radical3 x radical3)R30 degrees-Sn/Pt(111) surface alloy. *Langmuir : the ACS journal of surfaces and colloids* **2005**, *21* (3), 971–975. DOI: 10.1021/la048035q.

(31) Schwaab, V.; Hemauer, F.; Freiberger, E. M.; Waleska-Wellnhofer, N. J.; Steinrück, H.-P.; Papp, C. Liquid Organic Hydrogen Carriers: Model Catalytic Studies on the Thermal Dehydrogenation of 1-Cyclohexylethanol on Pt(111). *J. Phys. Chem. C* **2023**, *127* (23), 11058–11066. DOI: 10.1021/acs.jpcc.3c01969.

(32) Chen, J. J.; Winograd, N. The adsorption and decomposition of methylamine on Pd{111}. *Surface Science* **1995**, *326* (3), 285–300. DOI: 10.1016/0039-6028(94)00817-5.

(33) Lindquist, J. M.; Ziegler, J. P.; Hemminger, J. C. Photoelectron spectroscopy studies of the hydrogenation of cyanogen on Pt(111): Comparison with HCN and ethylenediamine. *Surface Science* **1989**, *210* (1-2), 27–45. DOI: 10.1016/0039-6028(89)90101-5.

(34) Ronning, C.; Feldermann, H.; Merk, R.; Hofsäss, H.; Reinke, P.; Thiele, J.-U. Carbon nitride deposited using energetic species: A review on XPS studies. *Phys. Rev. B* **1998**, *58* (4), 2207–2215. DOI: 10.1103/PhysRevB.58.2207.
